# Lessons from an eradication under multiple constraints of an island rat population of record density

**DOI:** 10.1111/cobi.70186

**Published:** 2025-12-02

**Authors:** Tatiane Micheletti, Thayná J. Mello, Carlos Verona, Vinícius P. O. Gasparotto, Ricardo Krul, Ricardo Araujo, Thali Sampaio, Paulo Rogerio Mangini

**Affiliations:** ^1^ TU Dresden Institute of Forest Growth and Computer Sciences Dresden Germany; ^2^ Faculty of Forestry, Forest Resources Management The University of British Columbia Vancouver British Columbia Canada; ^3^ Brazilian Institute for Conservation Medicine – TRIADE Recife Brazil; ^4^ Chico Mendes Institute for Biodiversity Conservation ICMBio Alcatrazes São Sebastião Brazil; ^5^ Oswaldo Cruz Foundation (Fiocruz) Rio de Janeiro Brazil; ^6^ Pontifical Catholic University (PUC) Rio de Janeiro Brazil; ^7^ Chico Mendes Institute for Biodiversity Conservation ICMBio Fernando de Noronha Fernando de Noronha Brazil

**Keywords:** adaptive management, biodiversity conservation, cost‐effective conservation strategies, invasive species management, native species conservation, rat eradication, real‐time monitoring, tropical islands, conservación de la biodiversidad, conservación de especias nativas, erradicación de ratas, estrategias rentables de conservación, gestión de especies invasoras, islas tropicales, manejo adaptativo, monitoreo en tiempo real, 入侵物种管理, 灭鼠, 热带岛屿, 适应性管理, 生物多样性保护, 实时监测, 原生物种保护, 具有成本效益的保护策略

## Abstract

Invasive rats threaten island biodiversity, disrupting ecosystems and endangering native species. Although rat eradication has succeeded on many islands, tropical islands present unique management challenges. Strict regulations and financial constraints on some tropical islands further limit proven eradication methods, complicating rodent management. We applied a real‐time active adaptive management approach that provided a cautious, cost‐efficient, and scientifically grounded pathway to rat eradication, while adhering to strict environmental regulations, on Ilha do Meio, Brazil. The cost was US$3300 per hectare, and the management actions were grounded in close interdisciplinary collaboration. We applied rodenticide (brodifacoum), monitored the rat population, and made iterative management adjustments. The rat overpopulation was eradicated within 5 months, and population increases were observed early on in the threatened masked booby (*Sula dactylatra*), and the endemic Noronha elaenia (*Elaenia ridleyana*) and Noronha skink (*Trachylepis atlantica*). Despite logistical constraints, our approach proved effective and cost‐efficient, marking its first application in a biological system. Our findings highlight the value of innovation, close interdisciplinary collaboration, and adaptive decision‐making when the application of best‐practice methods is constrained.

## Abstract (es)

Aprendizajes a partir de la erradicación bajo varias restricciones de una población insular de ratas con récord de densidad

**Resumen:**

Las ratas invasoras amenazan la biodiversidad de las islas, pues alteran los ecosistemas y ponen en peligro a las especies nativas. Aunque la erradicación de las ratas ha tenido éxito en muchas islas, las islas tropicales presentan retos de gestión únicos. Las estrictas regulaciones y las restricciones financieras en algunas islas tropicales limitan aún más los métodos de erradicación probados, lo que complica el manejo de los roedores. Aplicamos un enfoque de manejo adaptativo activo en tiempo real que proporcionó una vía cautelosa, rentable y con base científica para la erradicación de las ratas, al tiempo que se cumplían las estrictas regulaciones ambientales en Ilha do Meio, Brasil. El costo fue de 3,300 dólares estadounidenses por hectárea y las medidas de gestión se basaron en una estrecha colaboración interdisciplinaria. Aplicamos raticida (brodifacoum), supervisamos la población de ratas y realizamos ajustes de gestión iterativos. La superpoblación de ratas se erradicó en cinco meses y observamos un aumento poblacional de la sula enmascarada amenazada (*Sula dactylatra*) y de las especies endémicas el fiofío de Noronha (*Elaenia ridleyana*) y el lagarto de Noronha (*Trachylepis atlantica*). A pesar de las limitaciones logísticas, nuestro enfoque demostró ser eficaz y rentable, lo que supuso su primera aplicación en un sistema biológico. Nuestros hallazgos resaltan el valor de la innovación, la estrecha colaboración interdisciplinaria y la toma de decisiones adaptativas cuando la aplicación de los métodos de mejores prácticas se ve limitada.

## 在多重限制条件下清除密度创纪录的岛屿鼠群的经验教训

**【摘要】** 外来鼠类威胁着岛屿生物多样性, 破坏生态系统, 并危及原生物种。虽然许多岛屿的灭鼠工作都取得了成功, 但热带岛屿却面临着独特的管理挑战。一些热带岛屿严格的法规和财政限制进一步阻碍了公认有效的灭鼠方法的实施, 导致啮齿动物管理变得十分复杂。我们在巴西Ilha do Meio岛采用了一种实时、主动、适应性的管理方法, 在遵守严格的环境法规的同时, 提供了一种谨慎、经济高效、有科学依据的灭鼠方法。该方法每公顷的成本为3300美元, 管理行动以密切的跨学科合作为基础。我们施用了杀鼠剂(溴鼠灵), 监测鼠群数量, 并反复调整管理措施。鼠类种群过度增长的问题在5个月内得到了解决, 且在早期就观察到了濒危的蓝脸鲣鸟(*Sula dactylatra*)、当地特有的诺尔拟霸鹟(*Elaenia ridleyana*)和诺罗尼亚石龙子(*Trachylepis atlantica*)的种群数量增加。尽管存在安排协调方面的限制, 但我们的方法被证明是有效且高成本效益的, 这标志着它在生物系统中的首次应用。我们的研究结果凸显了在应用最佳实践方法受到限制时, 创新、密切的跨学科合作, 以及适应性决策的价值。**【翻译:胡怡思;审校:聂永刚】**

## INTRODUCTION

Biodiversity conservation faces growing threats from climate change to resource overexploitation (Rands et al., 2010). On islands, invasive species are a major challenge. They spread rapidly and cause severe ecological disruptions (Blackburn, 2004) that threaten local economies, public health, and cultural integrity (Nghiem et al., 2013). Invasive species are also closely linked to global biodiversity decline (Blackburn, 2004; Vitousek et al., 1997). Among them, black rats (*Rattus rattus*) are particularly damaging due to their adaptability and high reproductive rates (Doherty et al., 2016; Towns et al., 2006). They prey on native species, compete for resources, and disrupt entire ecosystems, driving biodiversity loss (Duron et al., 2017; Fukasawa et al., 2013; Graham et al., 2018; Harper & Bunbury, 2015; Quiterie et al., 2019; Tabak et al., 2014; Towns et al., 2009). However, rat eradication for biodiversity conservation has proven feasible and highly effective on islands (Holmes et al., 2019).

Successful rat eradications have been documented worldwide, from Alaska (Kurle et al., 2021) to New Zealand (Russell & Broome, 2016), although most have occurred in subtropical (e.g., Howald et al., 2010) and temperate regions (e.g., Capizzi, 2020). Despite notable exceptions (Griffiths et al., 2019; Samaniego‐Herrera et al., 2018), black rat eradications on tropical islands are less common, and failure rates are 2−2.4 times higher than in temperate zones (Russell & Holmes, 2015). Ecologically, tropical conditions favor invasive species by providing year‐round resources, mild winters (Holmes et al., 2015), and reduced bait exposure due to competition with native fauna (Wegmann et al., 2011). Socioeconomic barriers, including high costs, limited expertise, weak collaboration, and regulatory constraints, further hinder success (Samaniego et al., 2021). Although strict adherence to best practices improves outcomes (Samaniego et al., 2021), financial and regulatory limitations may prevent their full implementation.

Overcoming these challenges requires innovative approaches that account for ecological, social, and economic complexities while maximizing eradication success. Active adaptive management, or simply adaptive management, has emerged as a crucial strategy for improving conservation decisions (Chadès et al., 2017). Despite varying definitions (Månsson et al., 2023; Rist et al., 2013; Westgate et al., 2013; Williams & Brown, 2014), its core principle remains the integration of learning into management (McCarthy & Possingham, 2007; Chadès et al., 2017; Duron et al., 2017; McDonald‐Madden et al., 2010). Active adaptive management follows an iterative 6‐step process (modified from Westgate et al., 2013): define clear management goals; select multiple strategies; measure system responses; implement actions; monitor outcomes continuously; and adjust strategies accordingly. Incorporation of new information is ongoing, which reduces uncertainties that could hinder eradication.

With the rapid ecosystem changes in recent decades, real‐time monitoring has been increasingly integrated into adaptive management; thus, we define this process as real‐time adaptive management (RAM). Although RAM has advanced in automated fields, such as water (Rao et al., 1992) and soil management (Park & Harmon, 2011), as well as epidemiology (Atkins et al., 2020), it remains underdeveloped in ecology and biological systems. Despite its recognized value, adaptive management is rarely implemented in ecological projects, a trend persisting for over a decade (Månsson et al., 2023; Rist et al., 2013; Westgate et al., 2013).

We conducted an eradication project aimed at a dense population of black rats on a tropical island. We applied RAM to overcome ecological, financial, and regulatory constraints on the island. To our knowledge, ours is the first comprehensive application of adaptive management (following the scientific structure proposed by Westgate et al., 2013) and the first to document real‐time monitoring in a biological system. By detailing each step of the RAM framework and the challenges encountered, we sought to provide a practical model for invasive species management where best practices are difficult to implement. Close collaboration among field researchers, data analysts, and local managers was central to the project's success. We aimed to demonstrate how applied adaptive decision‐making and cautious innovation can offer insights for future conservation and policy development.

## METHODS

### Study area and species

**FIGURE 1:**
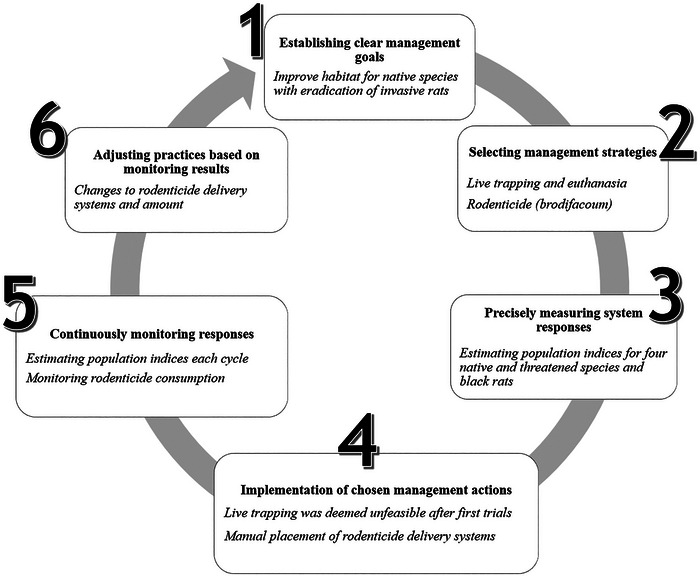
A scheme depicting our adaptive management protocol following the 6 iterative steps until objectives are met (modified from Westgate et al., 2013) (bold, step description; italics specific actions taken at each step).

Ilha do Meio, part of the Fernando de Noronha (FN) archipelago (3°50′41″S, 32°25′36″W), is an 18‐ha uninhabited island of high ecological importance that is free from human infrastructure (Appendix S1 & Figure 1a). Comprising 21 islands and islets and located 345 km off Brazil's northeastern coast, this volcanic archipelago is a UNESCO (United Nations Educational, Scientific and Cultural Organization) Natural World Heritage site due to its importance as a breeding and feeding area for seabirds and endemic species. Ilha do Meio hosts the endangered insular land crab (*Johngarthia lagostoma*); breeding populations of the masked (*Sula dactylatra*), brown (*Sula leucogaster*); endangered red‐footed booby (*Sula sula*); and white‐tailed tropicbird (*Phaethon lepturus*). The island also supports the endemic Noronha elaenia (*Elaenia ridleyana*), endangered Noronha skink, locally known as mabuya (*Trachylepis atlantica*), and Ridley's worm lizard (*Amphisbaena ridleyi*). Neighboring island Ilha Rata, a larger island 175 m away, separated by a strong tidal channel, shares similar characteristics and was used as a control site for wildlife monitoring. Although eradication was limited to Ilha do Meio, we planned monitoring focused on the land crab, Noronha elaenia, mabuya, white‐tailed tropicbird, and 3 booby species on both islands.

Fernando de Noronha has a warm climate, with water temperatures averaging 27°C and air temperatures ranging from 25°C to 31°C. Annual rainfall of 1400 mm occurs mainly from January to July. A drier season extends from August to December (WeatherSpark, 2024). The vegetation is classified as a seasonal deciduous forest and differs between the wet and dry seasons (Teixeira & Linsker, 2003). Despite its protected status in the National Marine Park of Fernando de Noronha, the archipelago's biodiversity faces ongoing threats from invasive species, including black (*R. rattus*) and brown rats (*R. norvegicus*), house mice (*Mus musculus*), domestic cats (*Felis silvestris catus*), dogs (*Canis lupus familiaris*), cururu toads (*Rhinella jimi*), and tegu lizards (*Salvator merianae*) (Micheletti et al., 2021).

### Hypotheses

We hypothesized time since eradication (TSE) starts to be a key predictor of native species’ population growth models when rat eradication has a significant impact on native species. We expected growth rates to increase over time, indicating positive effects on species recovery, and anticipated a corresponding positive trend in population growth when applying RAM.

### Adaptive management protocol

Our RAM protocol (Figure 1) was built through close interdisciplinary collaboration, a pioneering partnership among the National Marine Park of Fernando de Noronha (ICMBio), the World Wildlife Fund (WWF)‐Brazil, and Instituto Tríade to shape the project's adaptive design. Our RAM was based on recommendations from Wegmann et al. (2011), Westgate et al. (2013), and Keitt et al. (2015). It follows the 6 key steps of adaptive management (Westgate et al., 2013). The detailed protocol is in Appendix S1. Each cycle—full iteration of the RAM (Figure 1)—followed 3 iterative steps: first, rodenticide application and consumption monitoring; second, continuous monitoring of black rat and native wildlife populations; and third, management adjustments based on findings. Modifications resulting from our use of RAM are detailed in “Results.”

The primary management goal was to improve habitat for the endangered insular land crab, endemic Noronha elaenia and mabuya, threatened white‐tailed tropicbird, and 3 booby species. Although not monitored, the endemic Ridley's worm lizard was also expected to benefit. To achieve this, we aimed to eradicate black rats, the primary threat to native species, from Ilha do Meio. The project's US$60,000 budget was justified by its potential to prevent endemic species loss and protect seabird breeding grounds. This goal remained unchanged throughout the iterative adaptive management process.

We initially proposed 4 management strategies: live trapping followed by euthanasia and rodenticide application via aerial broadcast, manual deployment, and bait stations. The rodenticide used was brodifacoum (Table 1) and was chosen for its effectiveness and low failure rate (Parkes et al., 2011). Importantly, brodifacoum is the only rodenticide authorized by governmental regulatory agencies for use in the archipelago. Preliminary calculations estimated that live trapping would require at least 3 months of daily effort with 20 traps (Russell et al., 2018) based on the assumptions that rat densities were similar to those on the main island, there were no trap‐shy individuals, the weather was favorable, and there was no bait competition with land crabs. For rodenticide‐based strategies, we estimated that rats would need to consume 9 kg of bait. After presenting the initial plan to funders and the local park managers, we faced regulatory, logistical, financial, and ecological constraints and had to eliminate 3 of the 4 proposed strategies. Only the manual placement of brodifacoum bait blocks in bait stations was approved for implementation.

**TABLE 1:** Iterative cycles in the adaptive management framework applied to an eradication of black rats on the Ilha do Meio on the Fernando de Noronha archipelago (Brazil).^a^

	Description	Planning	Cycle 1	Cycle 2	Cycle 3	Cycle 4	Cycle 5
Campaign information	Objective		Method testing	Eradication effort	Eradication effort	Eradication confirmation	Eradication maintenance
	Month		August	October	December	January	April
	Year		2017	2017	2017	2018	2018
	Season		Wet	Dry	Dry	Dry	Wet
Capture‐recapture method	Number of traps		16	20	Not performed	25	10
	Distance between traps (m)		5.6–180^b^	12		50	80
	Trapping days		5	6		13	4
	Trapping effort (hours)^c^		1920	2880		4920	960
	Rat density (n/ha)	49	540	3.5		0	0
	Home range index (m)	7	2.8	6		0	0
Rodenticide application	Number of baiting stations	72	315	360	360	180	42
	Bait station distance (m)	50	20	20	20	50	10−97 m
	Bait quantity (kg)	9	94.5	80	80	60	14
	Delivery method	blocks Brodifacoum	blocks Brodifacoum	pellets of Brodifacoum in reversed bottle	pellets of Brodifacoum in reversed bottle	pellets of Brodifacoum in reversed bottle	blocks Brodifacoum in PVC tube, one end closed

^a^ Tomahawk galvanized wire traps that were 450 × 210 × 210 mm, foldable, and had a hooked trigger were used for live trapping. The bait (KLERAT Mata Ratos, Syngenta Brasil) set in block format weighed 20 g and contained approximately 1 mg of rodenticide. Baited camera traps were deployed on May 26, 2023, for 15 days. Eradication success was tested through 1080 h of sampling across all vegetation types.

^b^ A case of 2 × 2 cluster grids of 5.6 m; distance between clusters 10−80 m.

^c^ Due to high diurnal rat activity and logistical constraints preventing multiple field visits per day, trapping effort was measured in trap hours rather than trap nights to more accurately reflect total exposure time and capture probability.

To measure the system's responses (i.e., habitat improvement), we used fixed‐radius point counts for the Noronha elaenia, insular land crab, and mabuya. Seabirds were monitored through simple census surveys. Black rat density was estimated using a spatially explicit capture‐recapture method with 16 live traps (Appendix S3). Although 24 traps were planned, 8 malfunctioned at deployment. Trap placement and numbers were adjusted based on preliminary results from the first cycle of the RAM (cycle 1). Detailed data and analysis methods are available from https://github.com/tati‐micheletti/analysisIlhaDoMeio.

Regarding the implementation of management actions, baiting was initially planned for twice‐weekly replenishment in 72 stations placed 50 m apart based on rat home‐range estimates (Ringler et al., 2014; Robinson & Dick, 2020). Rodenticide application was scheduled for the driest month (Samaniego‐Herrera et al., 2014), and monitoring was aligned accordingly. All procedures adhered to Brazil's animal welfare standards (CFMV, 2012), and the authorization for management was granted by ICMBio (SISBIO number 38804‐9).

Continuous monitoring was used to assess the effects of black rat eradication on native species and ensure there were no subsequent negative impacts from rodenticide, following the precautionary principle. Initially, 3 iterations of rodenticide application and monitoring were planned to refine methods. Continuous monitoring included bait availability checks, wildlife monitoring, and black rat capture‐recapture efforts.

Rodenticide application and black rat monitoring were adjusted in real time based on observations. Native species monitoring remained consistent but was extended for the Noronha elaenia due to new data from parallel projects. Eradication confirmation was initially planned for 2 years after the last sighting of rats at baited camera traps.

### Native and endemic species data analyses

We used an open N‐mixture population model (R package unmarked [Kellner et al., 2023]) to analyze wildlife populations and tested 45 model formulations (Appendix S1). All model formulations are in Appendix S2. Population growth predictions from the best models for Ilha do Meio were adjusted by subtracting those for Ilha Rata to create the differential population growth index (DPGI) to isolate treatment effects from regional factors (e.g., extreme events, natural cycles, breeding seasonality). A linear model of DPGI over time was then fitted to assess native species’ population growth trends.

Originally, we planned to survey all 3 booby species and the tropicbird on both islands. However, the first expedition revealed that the main nesting sites of the tropicbird, red‐footed booby, and brown booby on Ilha Rata were inaccessible. Additionally, not all tropicbird nests on Ilha do Meio were reachable. Due to this lack of comparable control data, these species were excluded from modeling.

### Use of artificial intelligence

We used SCISPACE (https://typeset.io/) and ResearchRabbit (https://researchrabbitapp.com/home) for literature searches. SCISPACE queries included “black rat eradication on islands,” “adaptive management of invasive species,” and related terms. Manuscripts were selected based on title and abstract screening, similar to Google Scholar searches. To ensure comprehensive coverage, key references (Rist et al., 2013; Samaniego‐Herrera et al., 2018; Westgate et al., 2013) were added to ResearchRabbit, which allowed for citation‐based exploration. ChatGPT 4.0 was used for sentence refinement.

## RESULTS

**FIGURE 2:**
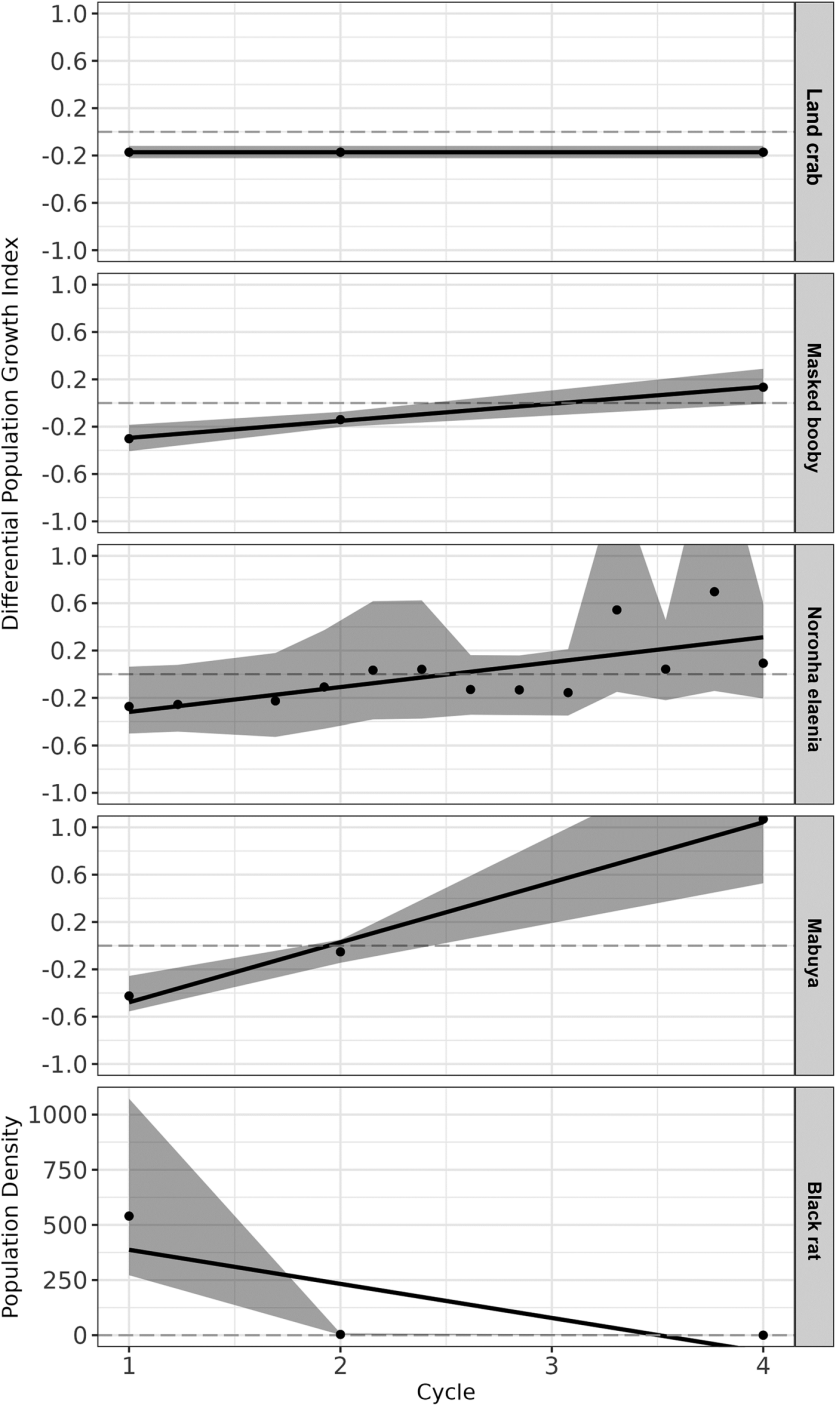
Differential population growth index values for 4 native species (land crab, masked booby, Noronha elaenia, and mabuya) across 4 cycles of black rat eradication with real‐time adaptive management and the population density of black rats (shading, confidence intervals; solid lines, population growth trends over time). The values for Noronha elaenia were rescaled from 15 to 4 cycles due to data availability.

The RAM project had a total direct cost of approximately US$60,000 (R$190,000 in 2017), or US$3300 per ha. Results are presented chronologically to illustrate the RAM process (Figure 1). A summary of the planning phase and the 5 iterative cycles is presented in Table 1. Adjustments made to the eradication cycle and wildlife and black rat monitoring results are detailed in Appendix S3. Over time, native species DPGI increased for all species except land crabs, and black rat density was reduced to zero within 3−4 cycles (Figure 2). All model objects and tables are available from https://zenodo.org/records/13830468.

### Management cycles

During cycle 1 (August 2017), when we deployed rodenticide bait stations (Appendix S3), the capture‐recapture trap grid revealed an unexpectedly high rat density (540 rats/ha). This prompted immediate project adjustments: increased rodenticide volume (additional 97 kg), increased number of bait stations (315 additional stations), and reduced station spacing (30 m less). Field observations indicated problems with mold and bait competition from crabs. During cycle 2 (October 2017), when brodifacoum pellets were introduced in a reversed bottle system (Appendix S3), rat density dropped substantially from 540 to 3.5 rats/ha. An additional 80 kg of brodifacoum was distributed across 360 bait stations. During cycle 3 (December 2017), we focused exclusively on bait replenishment due to logistical constraints. In cycle 4 (January 2018), no rats were detected despite increased trapping effort, so we reduced the number of bait stations by 25%. This suggested eradication was achieved within 5 months. In cycle 5 (April 2018), efforts shifted to preventing reinvasion by reducing bait stations (*n* = 42) and establishing a *cordon sanitaire* with a new delivery method (Appendix S3). No rats were photographed or captured after this, confirming eradication success.

### Native and endemic species models and population parameters

TSE started significantly increased population growth for masked booby (mean = 0.14 [SE 0.0380], *p* = 0.0002) and mabuya (mean = 0.46 [0.0971], *p =* 1.87e‐06) on Ilha do Meio, with a positive but not significant trend for Noronha elaenia (mean = 0.22 [0.1343], *p =* 0.0933) (Appendix S3). Positive population growth trends were observed for masked booby (mean = 0.14 [0.0059], *p =* 0.0263), mabuya (mean = 0.51 [0.047, *p =* 0.058), and Noronha elaenia (mean = 0.21 [0.069], *p =* 0.011). Land crabs showed no TSE effects or significant DPGI trends. These results highlight the role of eradication and environmental conditions in species recovery and stability. The best models selected are presented in Appendix S3, together with all population densities, detection probabilities, and growth rates for both islands based on the best models, each in a separate table.

## DISCUSSION

We demonstrated the successful application of RAM to eradicate a highly dense black rat population. Eradication led to immediate biodiversity benefits; that is, rapid population growth for the endemic mabuya and Noronha elaenia, and for the threatened masked booby. In contrast, land crab populations appeared unaffected by rat removal. To our knowledge, this is the first implementation of RAM for invasive species eradication in a tropical region, the first rat eradication on a Brazilian island, and the first detailed RAM case study applied to a biological system. By providing a comprehensive account of this process, we aimed to offer a valuable reference for future conservation efforts.

### Lessons learned about adaptive management and the RAM approach

Adaptive management has been widely discussed over the past half‐century, but a persistent gap exists between theory and practice. Of 1336 published adaptive management studies, fewer than 1% included monitoring data (Westgate et al., 2013). Similarly, whereas 56% of evaluated studies (*n* = 187) advocated for adaptive management, fewer than 5% reported successful implementation, and none quantified benefits or costs (Rist et al., 2013). This trend extends to invasive species management; fewer than 3% of 118 studies mentioned invasive species and adaptive management, and provided implementation details (Appendix S4). Only 1 study incorporated real‐time learning (Sciarretta et al., 2016), despite not achieving eradication. Moreover, none of these studies were conducted in the tropics, where eradication faces unique ecological and logistical challenges (Samaniego et al., 2020; Samaniego‐Herrera et al., 2018).

A key barrier to adaptive management adoption is the misunderstanding of its application (Rist et al., 2013). The scientific definition of adaptive management (Westgate et al., 2013) requires advanced scientific structuring and data analyses, skills often lacking among land managers, especially in underfunded tropical regions, where conservation staff receive limited training (Passell, 2000). This raises concerns about how scientific adaptive management methods can be effectively implemented when managers lack the ability to assess success indicators. For example, the Merri Creek revegetation study in Australia (McCarthy & Possingham, 2007) is a textbook case of adaptive management in which Bayesian modeling and stochastic dynamic programming were used to optimize management strategies. Such approaches require specialized training and stable funding, conditions rarely met in tropical conservation programs. Until budget stability and manager training improve, adaptive management will likely remain confined to well‐funded regions.

Our RAM framework was effective in achieving eradication despite logistical, financial, ecological, and regulatory constraints, mainly due to a combination of the real‐time iterative adjustments and close transdisciplinary collaborations (detailed below). The approach enabled complete eradication within 5 months while allowing for continuous monitoring of native species.

### Lessons learned about monitoring wildlife during rat eradication efforts

On Ilha Rata, land crab observations are positively influenced by rainfall and negatively by moonlight (Hartnoll et al., 2006). We found that rats had no apparent impact on crab populations, which is consistent with results of other studies (Gaiotto et al., 2020). Even though crab mortality risk due to brodifacoum poisoning was low (Masuda et al., 2015; Pain et al., 2000), crabs consumed rodenticide. Therefore, secondary poisoning in humans warrants investigation, given the possible ongoing poaching of crabs. Brodifacoum was undetected in crab claws and body tissues 1 month after exposure, but was present in the first month (Pain et al., 2000). For masked boobies, rat eradication significantly boosted population growth on Ilha do Meio, as confirmed by DPGI results. On Ilha Rata, populations remained stable despite rats, which is consistent with studies showing booby persistence under predation (Bolton et al., 2011; Gouvêa & Mello, 2017). However, rats disturb incubating adults, which may lead to nest abandonment (Priddel et al., 2005), a hypothesis supported by our findings.

Regarding endemic fauna, rat eradication greatly increased mabuya population growth (DPGI results). Models showed higher initial densities in treed areas, reflecting their habitat use. On the main island, rats consume mabuya as 30.3% of their diet (Gaiotto et al., 2020). This consumption is likely higher on Ilha do Meio due to the lack of human waste. Some mabuya ingested rodenticide, but monitoring showed no adverse effects. Further studies should be conducted to confirm this. For the Noronha elaenia, population growth was negatively linked to rainfall, likely due to its reliance on insect availability in the early rainy season (Licarião & De Brito Silva, 2024). However, rat eradication significantly benefited population growth, as confirmed by DPGI trends. High variability in initial data suggested the need for supplementation, which was enabled by an independent monitoring project. Although dispersal among islands is biologically possible, evidence suggests high site fidelity: Noronha elaenia occurs on only 3 of the 26 islands in the archipelago (Licarião & De Brito Silva, 2024). Capture‐recapture and genetic studies could confirm this.

Wildlife monitoring proved essential to monitoring the effects of the eradication and its unwanted consequences, and detecting population trends by the final eradication cycle (cycle 4). This underscores the value of real‐time monitoring in conservation decisions.

### Lessons learned about close collaboration

The project's success was heavily reliant on interdisciplinary collaboration between local environmental agencies, conservation practitioners, and scientific researchers. Close coordination facilitated rapid decision‐making, particularly in adjusting strategies based on real‐time data. This initiative was a pioneering collaboration between ICMBio, WWF‐Brazil, and Instituto Tríade, marking a milestone in marine protected area management in Brazil. Historically, Brazil has lacked expertise in invasive species eradication (Sampaio & Schmidt, 2013), and there have been only a few large‐scale management efforts (Fonseca et al., 2024; Silva & Alves, 2011). Major invasive species threats remain unmanaged, such as the European wild boar (*Sus scrofa*), one of the world's 100 most invasive species (Lowe et al., 2000). Wild boars now occur across all 6 Brazilian biomes (Fonseca et al., 2024), and despite their being targeted by the largest national invasive species control initiative, no strategies have been effective (Kmetiuk et al., 2023). Our project highlights the power of close interdisciplinary collaboration in adaptive management projects (Dreiss et al., 2017), setting a precedent for future invasive species management in Brazil.

### Lessons learned about handling the unexpected

Previous estimates for the main island of Fernando de Noronha suggest a mean rat density of 37 rats/ha (SE 12) (Russell et al., 2018), but there were no data for Ilha do Meio. Our first‐week estimate revealed an unprecedented 540 (195) rats/ha—11 times higher than the main island and exceeding all known records (64/ha in Hawaii [Tamarin & Malecha, 1971]; 66/ha in Mexico [Samaniego‐Herrera et al., 2018]; 187 rats/ha on Chagos Archipelago [95% CI 176−201] [Robinson & Dick, 2020; Vogt et al., 2014]). This triggered real‐time recalculations of rodenticide quantities, a challenge effectively addressed through the RAM approach. Although this extreme density may raise questions, anecdotal evidence (e.g., high day‐ and nighttime rat activity) long suggested an overpopulation, supported by high reproduction rates, abundant resources, and predator absence. Trap spacing of 25−50 m has been suggested to ensure bait encounter for black rats (Ringler et al., 2014), though ideally, spacing should be less than twice the home range scale (Sun et al., 2014). Shorter distances (∼10 m) improve density estimates in small areas (Sakamoto et al., 2015). Due to uncertainties, logistical constraints, and trap malfunctions, we used an irregular grid, which optimized detection probabilities at the expense of precision, an approach recently supported for similar cases (Durbach et al., 2021; Freeman et al., 2022). Although our model performed well, care should be taken when using irregular grids because they may introduce biases (Smith et al., 2020).

Declaring eradication success posed another challenge because no standardized protocol exists, and premature cessation has led to failures in past programs. Given uncertainties in detecting invasive species, eradication can rarely be guaranteed (Baker & Bode, 2021), making ongoing surveillance essential (Ramsey et al., 2009, 2011; Samaniego‐Herrera et al., 2013). To that effect, we expanded trapping effort and conducted ad hoc monitoring of rodenticide consumption and indirect rat signs (e.g., bite marks, footprints, feces, predation on seabirds) during cycle 4. Although 15–20 trap nights are often recommended for eradication confirmation (Russell et al., 2017), we set a conservative 2‐year window due to the significance of Brazil's first rat eradication. The unexpected pandemic delayed confirmation until 2023, but despite concerns about reinvasion, Ilha do Meio remained rat‐free 5 years after eradication. Although the island's proximity to Ilha Rata is within black rat swimming ability (Bagasara et al., 2016; King et al., 1974; Parkes et al., 2017; Shiels et al., 2014), empirical evidence suggests no genetic flux between the islands (Gatto‐Almeida et al., 2020).

### Lessons learned about balancing social perception, environmental safety, and efficiency

Although long‐term rodent control is challenging, live trapping followed by euthanasia has been effective for small areas (Duron et al., 2020) and is often preferred where rodenticides face social opposition (Duron et al., 2020; Fonseca et al., 2024). This option was considered but ultimately dismissed due to unlikely assumptions—including similar rat densities to the main island, absence of trap‐shy individuals, stable weather, and minimal bait competition from land crabs.

Although manual bait station placement was labor‐intensive when compared with aerial dispersal, strict regulations necessitated this approach. However, it had advantages. We used 18.25 kg/ha of rodenticide, which aligned with temperate (Broome et al., 2014) and dry tropical islands (Samaniego‐Herrera et al., 2018). Previous efforts on wet tropical islands have reported much higher bait usage (Harper et al., 2019; Samaniego‐Herrera et al., 2018; Wegmann et al., 2012), demonstrating the variability and potential for substantially higher requirements in tropical regions (Pott et al., 2015). Yet, our work aligned with previous observations that rat eradication on some mesic‐tropical islands may be achievable with typical temperate bait application rates (Griffiths et al., 2011).

Bait stations—while not the preferred approach among many practitioners—were designed within our precautionary RAM framework to detect potential negative impacts on native wildlife at the earliest stage. This was mandated by local authorities and was especially critical given that no similar rodent management had ever been conducted on islands in Brazil, particularly within a national park. In the event of negative effects (e.g., declining population trends in monitored native species), our approach would allow for immediate intervention, including the potential removal of georeferenced rodenticide baiting stations if necessary. Additionally, reduced bait consumption by land crabs—likely due to improved bait delivery systems, which also mitigated mold issues during the wet season—ensured greater bait availability for rats and led to effective lethal dosing across all rat territories (Howald et al., 2007) with reduced bait quantity.

### Lessons learned about combining policy and research

Our findings underscore the importance of designing adaptive strategies that account for ecological and regulatory contexts (Walters & Holling, 1990). The RAM framework proved effective in navigating these restrictions by integrating continuous monitoring and iterative adjustments (Williams, 2011)—enabling real‐time responses to emerging challenges. However, 2 key challenges must be addressed for long‐term effective policy making in conservation: lack of funding for research and awareness (Martin et al., 2012) and risk of excessive precaution reinforcing the status quo (Cooney, 2005).

Without adequate financial support for parallel research, adaptive strategies may struggle to gain traction and are more likely to fail. In our project, not all researchers were employed full‐time, and much of the work was conducted on a pro bono basis, an unfortunately common, yet unreported, situation in many developing countries. Although funding may cover equipment, consumables, and occasionally technical staff, there is a chronic lack of support for compensating researchers involved in management projects. As a result, research often becomes a secondary objective in management initiatives, if it is considered at all. This issue requires urgent attention from funding bodies and society because it may also contribute to the underrepresentation of adaptive management projects in peer‐reviewed literature, particularly in tropical regions (Martin et al., 2012).

To mitigate the reduced funding, we leveraged researchers’ field expertise in data collection and rodent management, allowing these tasks to be carried out simultaneously. For instance, concurrent research and management enabled real‐time coordination of bait replenishment with managers and field staff, ensuring bait availability until the next campaign based on observed consumption rates. This important integration of management and science (Runge, 2011; Williams, 2011) was crucial to the success of the RAM strategy, despite the reduced team size (6 researchers).

Although stringent regulations are designed to mitigate risks to nontarget species, they may also impose constraints that complicate the implementation of best practices in conservation, consequently allowing ecological degradation to persist (Cooney, 2005). In our project, both safety and environmental concerns regarding the proximity of nesting seabird colonies (i.e., risk of accident), and the potential necessity of poison removal influenced the decision to forgo aerial baiting. Although aerial distribution has proven highly effective in numerous island eradication programs (e.g., Harper et al., 2019; Wegmann et al., 2012), a cautious, site‐specific approach was deemed necessary to minimize unintended risks. Consequently, a labor‐intensive manual baiting strategy was employed, increasing logistical complexity and potentially extending the duration of rodent pressure on wildlife before eradication was achieved (Russell & Broome, 2016).

Although precautionary measures aim to minimize risks to nontarget species, they can also inadvertently hinder conservation efforts by delaying action and increasing the likelihood of eradication failure (Parkes et al., 2011). In our case, the decision to forgo aerial baiting in favor of a gradual, manual approach increased logistical complexity and potentially prolonged exposure risks before eradication was achieved. A more adaptive regulatory framework—one that integrates site‐specific risk assessments and controlled trials—could help balance safety and environmental concerns with the urgency of invasive species management and ensure effective and lower‐risk conservation outcomes.

The rapid recovery of habitat and biodiversity following rat eradication, as we observed, aligns with findings from other research on similar tropical islands (Miller‐ter Kuile et al., 2021). This highlights the potential for significant ecological restoration in relatively short timeframes, reinforcing the importance of timely and decisive management actions.

### Lessons learned about balancing risks

Our project had risks. Following best practices (Broome et al., 2014; Keitt et al., 2015) would have required conducting trials and assessments before eradication, ensuring bait exposure to all rats within 1–3 days—a key factor in reducing failure risks. Manually placed bait stations, while necessary due to regulatory constraints, increased the risk of incomplete exposure, which could have led to failure. However, delaying eradication would have jeopardized native species, making immediate action the best option. To mitigate risks, we leveraged real‐time monitoring and data science support, ultimately achieving successful eradication despite the odds.

Monitoring during eradication is generally discouraged due to potential confounding seasonal and environmental factors. However, it was essential for detecting adverse effects on native species and pausing interventions if necessary. By using Ilha Rata as a control site, we were able to isolate eradication effects and ensure observed changes on Ilha do Meio were attributable to rat removal rather than natural fluctuations.

One major limitation was the lack of monitoring for rodenticide fallout, posing potential risks to marine mammals and other nontarget fauna. Although brodifacoum residues were undetected 3 years post‐eradication at Palmyra Atoll (Wegmann et al., 2019), we strongly recommend future management actions secure funding to monitor potential environmental contamination and nontarget impacts.

Our study serves as a valuable example of conservation in a situation with multiple constraints, and our results showed that invasive species eradication is achievable without compromising ecological or legal safeguards. However, our experience underscores the need for policy reform and greater investment in conservation to tackle underlying challenges. Close collaboration among practitioners, policy makers, and researchers will be essential to the success of future eradication efforts, particularly in biodiversity‐rich tropical regions.

## Supporting information



Supporting Information

Supporting Information

Supporting Information

Supporting Information
