# Supplementary material for: Lessons from an eradication under multiple constraints of an island rat population of record density
Source: Conserv Biol. 2025 Dec 2;40(2):e70186. doi: 10.1111/cobi.70186 (PMC13036298; doi:10.1111/cobi.70186)
Supplement: Supplementary file 2 — Supporting Information [file COBI-40-e70186-s003.pdf]

Appendix S2. All model formulations tested for estimating population parameters of endangered and native species monitored on Ilha do Meio and Ilha Rata (Fernando de Noronha, Brazil) during an eradication attempt. We used open N-mixture population models from the R package unmarked (Kellner et al. 2023) to estimate lambda (initial population), gamma (population growth), det (probability of observation), as well as psi (zero-inflation) and alpha (dispersion) for models ZIP (zero inflated) and NB (negative binomial), respectively. Mixture P stands for Poisson and K for maximum number of individuals.

species	island	model	parameter	covariate	estimate	standardError	z	p	AIC	modelMixture	K
Trachylepis atlantica	Ilha do Meio	lam(.)gamma(.)p(.)iota(.)	lambda	(Intercept)	2.0188915	0.172029245	11.7357457	8.36E-32	697.4673604	ZIP	50
Trachylepis atlantica	Ilha do Meio	lam(.)gamma(.)p(.)iota(.)	gamma	(Intercept)	0.1253029	0.042348906	2.958823211	0.003088162	697.4673604	ZIP	50
Trachylepis atlantica	Ilha do Meio	lam(.)gamma(.)p(.)iota(.)	det	(Intercept)	-0.9247917	0.234802297	-3.938597538	8.20E-05	697.4673604	ZIP	50
Trachylepis atlantica	Ilha do Meio	lam(.)gamma(.)p(.)iota(.)	psi	psi	-1.1288439	0.374047414	-3.01791665	0.002545189	697.4673604	ZIP	50
Trachylepis atlantica	Ilha do Meio	lam(landscapeCover)gamma(.)p(.)iota(.)	lambda	(Intercept)	2.5777004	0.182657955	14.11217148	3.20E-45	627.9419974	ZIP	50
Trachylepis atlantica	Ilha do Meio	lam(landscapeCover)gamma(.)p(.)iota(.)	lambda	landscapeCoveropen	-2.5463677	0.394099033	-6.461238125	1.04E-10	627.9419974	ZIP	50
Trachylepis atlantica	Ilha do Meio	lam(landscapeCover)gamma(.)p(.)iota(.)	lambda	landscapeCoversemi-open	-1.2360641	0.375137145	-3.294965868	0.000984336	627.9419974	ZIP	50
Trachylepis atlantica	Ilha do Meio	lam(landscapeCover)gamma(.)p(.)iota(.)	lambda	landscapeCovertreed	0.4741232	0.176856338	2.680838217	0.007343801	627.9419974	ZIP	50
Trachylepis atlantica	Ilha do Meio	lam(landscapeCover)gamma(.)p(.)iota(.)	gamma	(Intercept)	0.0893472	0.039043013	2.288429289	0.022112534	627.9419974	ZIP	50
Trachylepis atlantica	Ilha do Meio	lam(landscapeCover)gamma(.)p(.)iota(.)	det	(Intercept)	-1.5100845	0.156042456	-9.677394809	3.76E-22	627.9419974	ZIP	50
Trachylepis atlantica	Ilha do Meio	lam(landscapeCover)gamma(.)p(.)iota(.)	psi	psi	-11.224923	65.75397008	-0.170710959	0.864451045	627.9419974	ZIP	50
Trachylepis atlantica	Ilha do Meio	lam(.)gamma(TSE)p(.)iota(.)	lambda	(Intercept)	2.1571117	0.17307814	12.46322441	1.18E-35	687.6418967	ZIP	50
Trachylepis atlantica	Ilha do Meio	lam(.)gamma(TSE)p(.)iota(.)	gamma	(Intercept)	0.1047958	0.042283776	2.478391945	0.013197608	687.6418967	ZIP	50
Trachylepis atlantica	Ilha do Meio	lam(.)gamma(TSE)p(.)iota(.)	gamma	scale(TSE)	0.2265352	0.065779431	3.443860494	0.000573472	687.6418967	ZIP	50
Trachylepis atlantica	Ilha do Meio	lam(.)gamma(TSE)p(.)iota(.)	det	(Intercept)	-0.9279988	0.209903387	-4.421076041	9.82E-06	687.6418967	ZIP	50
Trachylepis atlantica	Ilha do Meio	lam(.)gamma(TSE)p(.)iota(.)	psi	psi	-1.1268611	0.373334663	-3.018367229	0.002541407	687.6418967	ZIP	50
Trachylepis atlantica	Ilha do Meio	lam(landscapeCover)gamma(TSE)p(.)iota(.)	lambda	(Intercept)	2.6115667	0.1827154	14.29308497	2.42E-46	621.3222029	ZIP	50
Trachylepis atlantica	Ilha do Meio	lam(landscapeCover)gamma(TSE)p(.)iota(.)	lambda	landscapeCoveropen	-2.5064633	0.393937036	-6.362598682	1.98E-10	621.3222029	ZIP	50
Trachylepis atlantica	Ilha do Meio	lam(landscapeCover)gamma(TSE)p(.)iota(.)	lambda	landscapeCoversemi-open	-1.1852837	0.372953462	-3.178100853	0.001482432	621.3222029	ZIP	50
Trachylepis atlantica	Ilha do Meio	lam(landscapeCover)gamma(TSE)p(.)iota(.)	lambda	landscapeCovertreed	0.4477584	0.179633434	2.492622961	0.012680342	621.3222029	ZIP	50
Trachylepis atlantica	Ilha do Meio	lam(landscapeCover)gamma(TSE)p(.)iota(.)	gamma	(Intercept)	0.0727292	0.039705266	1.831725623	0.066992309	621.3222029	ZIP	50
Trachylepis atlantica	Ilha do Meio	lam(landscapeCover)gamma(TSE)p(.)iota(.)	gamma	scale(TSE)	0.1948583	0.06649655	2.930351639	0.003385786	621.3222029	ZIP	50
Trachylepis atlantica	Ilha do Meio	lam(landscapeCover)gamma(TSE)p(.)iota(.)	det	(Intercept)	-1.3770323	0.16184633	-8.508270432	1.77E-17	621.3222029	ZIP	50
Trachylepis atlantica	Ilha do Meio	lam(landscapeCover)gamma(TSE)p(.)iota(.)	psi	psi	-11.499047	75.82661384	-0.151649223	0.879463608	621.3222029	ZIP	50
Trachylepis atlantica	Ilha do Meio	lam(.)gamma(.)p(observerID)iota(.)	lambda	(Intercept)	2.7938867	0.254188356	10.99140319	4.20E-28	641.9758977	ZIP	50
Trachylepis atlantica	Ilha do Meio	lam(.)gamma(.)p(observerID)iota(.)	gamma	(Intercept)	0.0442353	0.049456988	0.894420254	0.37109708	641.9758977	ZIP	50
Trachylepis atlantica	Ilha do Meio	lam(.)gamma(.)p(observerID)iota(.)	det	(Intercept)	-4.1058258	0.30449908	-13.48386916	1.95E-41	641.9758977	ZIP	50
Trachylepis atlantica	Ilha do Meio	lam(.)gamma(.)p(observerID)iota(.)	det	observerIDMangini	2.7145967	0.244553385	11.10022145	1.25E-28	641.9758977	ZIP	50
Trachylepis atlantica	Ilha do Meio	lam(.)gamma(.)p(observerID)iota(.)	det	observerIDVerona	2.4926199	0.321862563	7.744360995	9.61E-15	641.9758977	ZIP	50
Trachylepis atlantica	Ilha do Meio	lam(.)gamma(.)p(observerID)iota(.)	det	observerIDVini	3.1155213	0.303664408	10.25975127	1.07E-24	641.9758977	ZIP	50
Trachylepis atlantica	Ilha do Meio	lam(.)gamma(.)p(observerID)iota(.)	psi	psi	-8.5060125	48.22771229	-0.176371884	0.860001792	641.9758977	ZIP	50
Trachylepis atlantica	Ilha do Meio	lam(landscapeCover)gamma(.)p(observerID)iota(.)	lambda	(Intercept)	2.8040126	0.201213194	13.93553062	3.85E-44	622.9282287	ZIP	50
Trachylepis atlantica	Ilha do Meio	lam(landscapeCover)gamma(.)p(observerID)iota(.)	lambda	landscapeCoveropen	-2.1178999	0.734193367	-2.88466225	0.003918338	622.9282287	ZIP	50
Trachylepis atlantica	Ilha do Meio	lam(landscapeCover)gamma(.)p(observerID)iota(.)	lambda	landscapeCoversemi-open	-0.741536	0.72730369	-1.019568594	0.307933106	622.9282287	ZIP	50
Trachylepis atlantica	Ilha do Meio	lam(landscapeCover)gamma(.)p(observerID)iota(.)	lambda	landscapeCovertreed	0.6351392	0.177039914	3.587548139	0.000333802	622.9282287	ZIP	50
Trachylepis atlantica	Ilha do Meio	lam(landscapeCover)gamma(.)p(observerID)iota(.)	gamma	(Intercept)	-0.0073791	0.049195226	-0.149996125	0.880767672	622.9282287	ZIP	50
Trachylepis atlantica	Ilha do Meio	lam(landscapeCover)gamma(.)p(observerID)iota(.)	det	(Intercept)	-2.3279913	0.791243523	-2.942193178	0.003258966	622.9282287	ZIP	50
Trachylepis atlantica	Ilha do Meio	lam(landscapeCover)gamma(.)p(observerID)iota(.)	det	observerIDMangini	0.6667573	0.810264939	0.822888011	0.410571681	622.9282287	ZIP	50
Trachylepis atlantica	Ilha do Meio	lam(landscapeCover)gamma(.)p(observerID)iota(.)	det	observerIDVerona	0.0883789	0.842277827	0.104928415	0.916432612	622.9282287	ZIP	50
Trachylepis atlantica	Ilha do Meio	lam(landscapeCover)gamma(.)p(observerID)iota(.)	det	observerIDVini	1.1269592	0.819288371	1.375534281	0.168965865	622.9282287	ZIP	50
Trachylepis atlantica	Ilha do Meio	lam(landscapeCover)gamma(.)p(observerID)iota(.)	psi	psi	-8.3530648	18.39920596	-0.453990503	0.649835662	622.9282287	ZIP	50
Trachylepis atlantica	Ilha do Meio	lam(.)gamma(TSE)p(observerID)iota(.)	lambda	(Intercept)	2.5933007	0.224534923	11.54965403	7.41E-31	632.2293933	ZIP	50
Trachylepis atlantica	Ilha do Meio	lam(.)gamma(TSE)p(observerID)iota(.)	gamma	(Intercept)	0.0661451	0.049546541	1.335010012	0.18187307	632.2293933	ZIP	50
Trachylepis atlantica	Ilha do Meio	lam(.)gamma(TSE)p(observerID)iota(.)	gamma	scale(TSE)	0.3246261	0.091328563	3.554485849	0.000378719	632.2293933	ZIP	50
Trachylepis atlantica	Ilha do Meio	lam(.)gamma(TSE)p(observerID)iota(.)	det	(Intercept)	-3.6724909	0.310093193	-11.84318448	2.33E-32	632.2293933	ZIP	50
Trachylepis atlantica	Ilha do Meio	lam(.)gamma(TSE)p(observerID)iota(.)	det	observerIDMangini	2.8566733	0.265044424	10.77809248	4.37E-27	632.2293933	ZIP	50
Trachylepis atlantica	Ilha do Meio	lam(.)gamma(TSE)p(observerID)iota(.)	det	observerIDVerona	2.2767908	0.336637667	6.763327546	1.35E-11	632.2293933	ZIP	50
Trachylepis atlantica	Ilha do Meio	lam(.)gamma(TSE)p(observerID)iota(.)	det	observerIDVini	2.5378953	0.342169657	7.417067086	1.20E-13	632.2293933	ZIP	50
Trachylepis atlantica	Ilha do Meio	lam(.)gamma(TSE)p(observerID)iota(.)	psi	psi	-9.8297195	77.87804185	-0.126219397	0.899558257	632.2293933	ZIP	50
Trachylepis atlantica	Ilha do Meio	lam(landscapeCover)gamma(TSE)p(observerID)iota(.)	lambda	(Intercept)	4.6346109	0.365235778	12.68936715	6.77E-37	587.7750969	ZIP	650
Trachylepis atlantica	Ilha do Meio	lam(landscapeCover)gamma(TSE)p(observerID)iota(.)	lambda	landscapeCoveropen	-3.5370469	0.836400261	-4.228892657	2.35E-05	587.7750969	ZIP	650
Trachylepis atlantica	Ilha do Meio	lam(landscapeCover)gamma(TSE)p(observerID)iota(.)	lambda	landscapeCoversemi-open	-2.1408288	0.818179026	-2.616577503	0.00888162	587.7750969	ZIP	650
Trachylepis atlantica	Ilha do Meio	lam(landscapeCover)gamma(TSE)p(observerID)iota(.)	lambda	landscapeCovertreed	1.0922221	0.153612311	7.110250868	1.16E-12	587.7750969	ZIP	650
Trachylepis atlantica	Ilha do Meio	lam(landscapeCover)gamma(TSE)p(observerID)iota(.)	gamma	(Intercept)	0.0092199	0.049945696	0.184598632	0.853543859	587.7750969	ZIP	650
Trachylepis atlantica	Ilha do Meio	lam(landscapeCover)gamma(TSE)p(observerID)iota(.)	gamma	scale(TSE)	0.4626561	0.097060217	4.766691193	1.87E-06	587.7750969	ZIP	650
Trachylepis atlantica	Ilha do Meio	lam(landscapeCover)gamma(TSE)p(observerID)iota(.)	det	(Intercept)	-2.4765179	0.795474267	-3.11325964	0.001850332	587.7750969	ZIP	650
Trachylepis atlantica	Ilha do Meio	lam(landscapeCover)gamma(TSE)p(observerID)iota(.)	det	observerIDMangini	-1.0501837	0.881040846	-1.191980743	0.233268796	587.7750969	ZIP	650
Trachylepis atlantica	Ilha do Meio	lam(landscapeCover)gamma(TSE)p(observerID)iota(.)	det	observerIDVerona	-2.1418172	0.883422705	-2.42445346	0.015331448	587.7750969	ZIP	650
Trachylepis atlantica	Ilha do Meio	lam(landscapeCover)gamma(TSE)p(observerID)iota(.)	det	observerIDVini	-1.400695	0.847839213	-1.652076259	0.098519006	587.7750969	ZIP	650
Trachylepis atlantica	Ilha do Meio	lam(landscapeCover)gamma(TSE)p(observerID)iota(.)	psi	psi	-12.979236	199.2236807	-0.065149063	0.948055317	587.7750969	ZIP	650
Elaenia ridleyana	Ilha do Meio	lam(.)gamma(.)p(.)iota(.)	lambda	(Intercept)	1.4982797	0.497237215	3.013209032	0.002585008	429.8515226	ZIP	50
Elaenia ridleyana	Ilha do Meio	lam(.)gamma(.)p(.)iota(.)	gamma	(Intercept)	-0.0041462	0.119047507	-0.034827901	0.972216972	429.8515226	ZIP	50
Elaenia ridleyana	Ilha do Meio	lam(.)gamma(.)p(.)iota(.)	det	(Intercept)	0.1138024	0.179810653	0.632901472	0.526797986	429.8515226	ZIP	50
Elaenia ridleyana	Ilha do Meio	lam(.)gamma(.)p(.)iota(.)	psi	psi	-8.1743722	59.58891349	-0.137179413	0.890888983	429.8515226	ZIP	50
Elaenia ridleyana	Ilha do Meio	lam(.)gamma(totalRainfallThreeMonths)p(.)iota(.)	lambda	(Intercept)	1.5502792	0.493159538	3.143565227	0.001669032	424.8159325	ZIP	50
Elaenia ridleyana	Ilha do Meio	lam(.)gamma(totalRainfallThreeMonths)p(.)iota(.)	gamma	(Intercept)	-0.0168349	0.119233317	-0.141193025	0.887717456	424.8159325	ZIP	50
Elaenia ridleyana	Ilha do Meio	lam(.)gamma(totalRainfallThreeMonths)p(.)iota(.)	gamma	scale(totalRainfallThreeMonths)	-0.3627573	0.139603774	-2.598477428	0.00936382	424.8159325	ZIP	50
Elaenia ridleyana	Ilha do Meio	lam(.)gamma(totalRainfallThreeMonths)p(.)iota(.)	det	(Intercept)	0.0173962	0.190701219	0.09122242	0.92731586	424.8159325	ZIP	50
Elaenia ridleyana	Ilha do Meio	lam(.)gamma(totalRainfallThreeMonths)p(.)iota(.)	psi	psi	-4.2080156	8.320928301	-0.505714676	0.613056964	424.8159325	ZIP	50
Elaenia ridleyana	Ilha do Meio	lam(.)gamma(TSE)p(.)iota(.)	lambda	(Intercept)	1.4981139	0.497286831	3.012575133	0.002590413	431.8523038	ZIP	50
Elaenia ridleyana	Ilha do Meio	lam(.)gamma(TSE)p(.)iota(.)	gamma	(Intercept)	-0.0042697	0.119061311	-0.035861766	0.971392583	431.8523038	ZIP	50
Elaenia ridleyana	Ilha do Meio	lam(.)gamma(TSE)p(.)iota(.)	gamma	scale(TSE)	-0.0025162	0.121782043	-0.020661162	0.983515951	431.8523038	ZIP	50
Elaenia ridleyana	Ilha do Meio	lam(.)gamma(TSE)p(.)iota(.)	det	(Intercept)	0.1140134	0.179771967	0.6342111	0.525943065	431.8523038	ZIP	50
Elaenia ridleyana	Ilha do Meio	lam(.)gamma(TSE)p(.)iota(.)	psi	psi	-7.0144645	33.38579227	-0.21010328	0.833587065	431.8523038	ZIP	50
Elaenia ridleyana	Ilha do Meio	lam(.)gamma(totalRainfallThreeMonths+TSE)p(.)iota(.)	lambda	(Intercept)	1.5996945	0.488569297	3.27424288	0.001059455	424.1631536	ZIP	250
Elaenia ridleyana	Ilha do Meio	lam(.)gamma(totalRainfallThreeMonths+TSE)p(.)iota(.)	gamma	(Intercept)	-0.0197285	0.11837442	-0.166661443	0.867636445	424.1631536	ZIP	250
Elaenia ridleyana	Ilha do Meio	lam(.)gamma(totalRainfallThreeMonths+TSE)p(.)iota(.)	gamma	scale(totalRainfallThreeMonths)	-0.4895144	0.156321484	-3.131459308	0.001739399	424.1631536	ZIP	250
Elaenia ridleyana	Ilha do Meio	lam(.)gamma(totalRainfallThreeMonths+TSE)p(.)iota(.)	gamma	scale(TSE)	0.2254219	0.134299852	1.67849669	0.093250176	424.1631536	ZIP	250
Elaenia ridleyana	Ilha do Meio	lam(.)gamma(totalRainfallThreeMonths+TSE)p(.)iota(.)	det	(Intercept)	-0.0396801	0.203834984	-0.194667592	0.845653184	424.1631536	ZIP	250
Elaenia ridleyana	Ilha do Meio	lam(.)gamma(totalRainfallThreeMonths+TSE)p(.)iota(.)	psi	psi	-5.3502023	14.58272089	-0.366886424	0.713703739	424.1631536	ZIP	250
Johngarthia lagostoma	Ilha do Meio	lam(.)gamma(.)p(.)iota(.)	lambda	(Intercept)	2.406531	0.111061312	21.66849065	4.07E-104	910.1529831	ZIP	50
Johngarthia lagostoma	Ilha do Meio	lam(.)gamma(.)p(.)iota(.)	gamma	(Intercept)	0.0973218	0.034005563	2.861938633	0.004210584	910.1529831	ZIP	50
Johngarthia lagostoma	Ilha do Meio	lam(.)gamma(.)p(.)iota(.)	det	(Intercept)	-0.6057501	0.144220952	-4.200153133	2.67E-05	910.1529831	ZIP	50
Johngarthia lagostoma	Ilha do Meio	lam(.)gamma(.)p(.)iota(.)	psi	psi	-1.681913	0.448038104	-3.753950765	0.000174069	910.1529831	ZIP	50
Johngarthia lagostoma	Ilha do Meio	lam(landscapeCover)gamma(.)p(.)iota(.)	lambda	(Intercept)	1.9298423	0.197991878	9.747078083	1.90E-22	872.3355903	ZIP	50
Johngarthia lagostoma	Ilha do Meio	lam(landscapeCover)gamma(.)p(.)iota(.)	lambda	landscapeCoveropen	0.3052958	0.221574321	1.377848378	0.168250106	872.3355903	ZIP	50
Johngarthia lagostoma	Ilha do Meio	lam(landscapeCover)gamma(.)p(.)iota(.)	lambda	landscapeCovertreed	1.1213494	0.202439812	5.539174211	3.04E-08	872.3355903	ZIP	50
Johngarthia lagostoma	Ilha do Meio	lam(landscapeCover)gamma(.)p(.)iota(.)	gamma	(Intercept)	0.0722943	0.034128991	2.118265199	0.034152614	872.3355903	ZIP	50
Johngarthia lagostoma	Ilha do Meio	lam(landscapeCover)gamma(.)p(.)iota(.)	det	(Intercept)	-0.7091459	0.143605005	-4.938169696	7.89E-07	872.3355903	ZIP	50
Johngarthia lagostoma	Ilha do Meio	lam(landscapeCover)gamma(.)p(.)iota(.)	psi	psi	-1.7668734	0.482865575	-3.659141353	0.000253062	872.3355903	ZIP	50
Johngarthia lagostoma	Ilha do Meio	lam(.)gamma(TSE)p(.)iota(.)	lambda	(Intercept)	2.9474461	0.577459177	5.104163601	3.32E-07	35591.15924	ZIP	50
Johngarthia lagostoma	Ilha do Meio	lam(.)gamma(TSE)p(.)iota(.)	gamma	(Intercept)	-0.1688717	620.2957487	-0.000272244	0.999782781	35591.15924	ZIP	50
Johngarthia lagostoma	Ilha do Meio	lam(.)gamma(TSE)p(.)iota(.)	gamma	scale(TSE)	-0.2198125	476.6008081	-0.000461209	0.999632009	35591.15924	ZIP	50
Johngarthia lagostoma	Ilha do Meio	lam(.)gamma(TSE)p(.)iota(.)	det	(Intercept)	-1.2005398	0.762302473	-1.574886431	0.115282661	35591.15924	ZIP	50
Johngarthia lagostoma	Ilha do Meio	lam(.)gamma(TSE)p(.)iota(.)	psi	psi	-2.506659	1.062046186	-2.360216596	0.018264268	35591.15924	ZIP	50
Johngarthia lagostoma	Ilha do Meio	lam(landscapeCover)gamma(TSE)p(.)iota(.)	lambda	(Intercept)	1.4466414	NA	NA	NA	35532.73727	ZIP	50
Johngarthia lagostoma	Ilha do Meio	lam(landscapeCover)gamma(TSE)p(.)iota(.)	lambda	landscapeCoveropen	0	NA	NA	NA	35532.73727	ZIP	50
Johngarthia lagostoma	Ilha do Meio	lam(landscapeCover)gamma(TSE)p(.)iota(.)	lambda	landscapeCovertreed	2.3613867	NA	NA	NA	35532.73727	ZIP	50
Johngarthia lagostoma	Ilha do Meio	lam(landscapeCover)gamma(TSE)p(.)iota(.)	gamma	(Intercept)	0.1073766	NA	NA	NA	35532.73727	ZIP	50
Johngarthia lagostoma	Ilha do Meio	lam(landscapeCover)gamma(TSE)p(.)iota(.)	gamma	scale(TSE)	0.1397423	NA	NA	NA	35532.73727	ZIP	50
Johngarthia lagostoma	Ilha do Meio	lam(landscapeCover)gamma(TSE)p(.)iota(.)	det	(Intercept)	0.266815	NA	NA	NA	35532.73727	ZIP	50
Johngarthia lagostoma	Ilha do Meio	lam(landscapeCover)gamma(TSE)p(.)iota(.)	psi	psi	-3.0647161	NA	NA	NA	35532.73727	ZIP	50
Johngarthia lagostoma	Ilha do Meio	lam(.)gamma(.)p(totalRainfallThreeMonths)iota(.)	lambda	(Intercept)	2.3090088	0.117736814	19.61161225	1.23E-85	796.7253839	ZIP	50
Johngarthia lagostoma	Ilha do Meio	lam(.)gamma(.)p(totalRainfallThreeMonths)iota(.)	gamma	(Intercept)	0.0649793	0.031387653	2.070219128	0.038431829	796.7253839	ZIP	50
Johngarthia lagostoma	Ilha do Meio	lam(.)gamma(.)p(totalRainfallThreeMonths)iota(.)	det	(Intercept)	-0.7025703	0.162996539	-4.310338516	1.63E-05	796.7253839	ZIP	50
Johngarthia lagostoma	Ilha do Meio	lam(.)gamma(.)p(totalRainfallThreeMonths)iota(.)	det	scale(totalRainfallThreeMonths)	0.7166021	0.07947727	9.016441213	1.94E-19	796.7253839	ZIP	50
Johngarthia lagostoma	Ilha do Meio	lam(.)gamma(.)p(totalRainfallThreeMonths)iota(.)	psi	psi	-1.7337217	0.468489201	-3.700665178	0.000215035	796.7253839	ZIP	50
Johngarthia lagostoma	Ilha do Meio	lam(landscapeCover)gamma(.)p(totalRainfallThreeMonths)iota(.)	lambda	(Intercept)	1.8644326	0.193248375	9.647856434	5.02E-22	742.3764327	ZIP	50
Johngarthia lagostoma	Ilha do Meio	lam(landscapeCover)gamma(.)p(totalRainfallThreeMonths)iota(.)	lambda	landscapeCoveropen	0.2776025	0.221747178	1.251887287	0.210610938	742.3764327	ZIP	50
Johngarthia lagostoma	Ilha do Meio	lam(landscapeCover)gamma(.)p(totalRainfallThreeMonths)iota(.)	lambda	landscapeCovertreed	1.3195858	0.201551457	6.547140951	5.86E-11	742.3764327	ZIP	50
Johngarthia lagostoma	Ilha do Meio	lam(landscapeCover)gamma(.)p(totalRainfallThreeMonths)iota(.)	gamma	(Intercept)	0.0337198	0.029972022	1.125042037	0.260571221	742.3764327	ZIP	50
Johngarthia lagostoma	Ilha do Meio	lam(landscapeCover)gamma(.)p(totalRainfallThreeMonths)iota(.)	det	(Intercept)	-1.007157	0.135435279	-7.436444684	1.03E-13	742.3764327	ZIP	50
Johngarthia lagostoma	Ilha do Meio	lam(landscapeCover)gamma(.)p(totalRainfallThreeMonths)iota(.)	det	scale(totalRainfallThreeMonths)	0.7167472	0.066645052	10.75469457	5.63E-27	742.3764327	ZIP	50
Johngarthia lagostoma	Ilha do Meio	lam(landscapeCover)gamma(.)p(totalRainfallThreeMonths)iota(.)	psi	psi	-2.0603563	0.614939694	-3.350501447	0.000806654	742.3764327	ZIP	50
Johngarthia lagostoma	Ilha do Meio	lam(.)gamma(TSE)p(totalRainfallThreeMonths)iota(.)	lambda	(Intercept)	2.2471509	NA	NA	NA	35540.15143	ZIP	50
Johngarthia lagostoma	Ilha do Meio	lam(.)gamma(TSE)p(totalRainfallThreeMonths)iota(.)	gamma	(Intercept)	-0.5738069	NA	NA	NA	35540.15143	ZIP	50
Johngarthia lagostoma	Ilha do Meio	lam(.)gamma(TSE)p(totalRainfallThreeMonths)iota(.)	gamma	scale(TSE)	-0.7468716	NA	NA	NA	35540.15143	ZIP	50
Johngarthia lagostoma	Ilha do Meio	lam(.)gamma(TSE)p(totalRainfallThreeMonths)iota(.)	det	(Intercept)	0.6365811	NA	NA	NA	35540.15143	ZIP	50
Johngarthia lagostoma	Ilha do Meio	lam(.)gamma(TSE)p(totalRainfallThreeMonths)iota(.)	det	scale(totalRainfallThreeMonths)	3.2694211	NA	NA	NA	35540.15143	ZIP	50
Johngarthia lagostoma	Ilha do Meio	lam(.)gamma(TSE)p(totalRainfallThreeMonths)iota(.)	psi	psi	-6.5565467	18.14051311	-0.361431163	0.717777156	35540.15143	ZIP	50
Johngarthia lagostoma	Ilha do Meio	lam(landscapeCover)gamma(TSE)p(totalRainfallThreeMonths)iota(.)	lambda	(Intercept)	1.6297786	NA	NA	NA	35512.60693	ZIP	50
Johngarthia lagostoma	Ilha do Meio	lam(landscapeCover)gamma(TSE)p(totalRainfallThreeMonths)iota(.)	lambda	landscapeCoveropen	0	NA	NA	NA	35512.60693	ZIP	50
Johngarthia lagostoma	Ilha do Meio	lam(landscapeCover)gamma(TSE)p(totalRainfallThreeMonths)iota(.)	lambda	landscapeCovertreed	1.6691827	NA	NA	NA	35512.60693	ZIP	50
Johngarthia lagostoma	Ilha do Meio	lam(landscapeCover)gamma(TSE)p(totalRainfallThreeMonths)iota(.)	gamma	(Intercept)	-0.3496019	NA	NA	NA	35512.60693	ZIP	50
Johngarthia lagostoma	Ilha do Meio	lam(landscapeCover)gamma(TSE)p(totalRainfallThreeMonths)iota(.)	gamma	scale(TSE)	-0.4550629	NA	NA	NA	35512.60693	ZIP	50

Johngarthia lagostoma	Ilha do Meio	lam(landscapeCover)gamma(TSE)p(totalRainfallThreeMonths)iota(.)	det	(Intercept)	1.3455574	NA	NA	NA	35512.60693	ZIP	50
Johngarthia lagostoma	Ilha do Meio	lam(landscapeCover)gamma(TSE)p(totalRainfallThreeMonths)iota(.)	det	scale(totalRainfallThreeMonths)	3.2920694	NA	NA	NA	35512.60693	ZIP	50
Johngarthia lagostoma	Ilha do Meio	lam(landscapeCover)gamma(TSE)p(totalRainfallThreeMonths)iota(.)	psi	psi	-9.0452127	NA	NA	NA	35512.60693	ZIP	50
Johngarthia lagostoma	Ilha do Meio	lam(.)gamma(.)p(moonPhase)iota(.)	lambda	(Intercept)	2.4179791	0.112050835	21.57930458	2.81E-103	909.5269788	ZIP	50
Johngarthia lagostoma	Ilha do Meio	lam(.)gamma(.)p(moonPhase)iota(.)	gamma	(Intercept)	0.0890451	0.034121118	2.609677179	0.00906277	909.5269788	ZIP	50
Johngarthia lagostoma	Ilha do Meio	lam(.)gamma(.)p(moonPhase)iota(.)	det	(Intercept)	-0.6232951	0.14613985	-4.265059291	2.00E-05	909.5269788	ZIP	50
Johngarthia lagostoma	Ilha do Meio	lam(.)gamma(.)p(moonPhase)iota(.)	det	scale(moonPhase)	0.1019113	0.062955945	1.618771534	0.105496429	909.5269788	ZIP	50
Johngarthia lagostoma	Ilha do Meio	lam(.)gamma(.)p(moonPhase)iota(.)	psi	psi	-1.6818634	0.447729995	-3.75642326	0.000172359	909.5269788	ZIP	50
Johngarthia lagostoma	Ilha do Meio	lam(landscapeCover)gamma(.)p(moonPhase)iota(.)	lambda	(Intercept)	1.9212729	0.197909083	9.707855939	2.79E-22	871.7484501	ZIP	50
Johngarthia lagostoma	Ilha do Meio	lam(landscapeCover)gamma(.)p(moonPhase)iota(.)	lambda	landscapeCoveropen	0.3478616	0.222533549	1.563187307	0.118008545	871.7484501	ZIP	50
Johngarthia lagostoma	Ilha do Meio	lam(landscapeCover)gamma(.)p(moonPhase)iota(.)	lambda	landscapeCovertreed	1.13462	0.202273736	5.60932951	2.03E-08	871.7484501	ZIP	50
Johngarthia lagostoma	Ilha do Meio	lam(landscapeCover)gamma(.)p(moonPhase)iota(.)	gamma	(Intercept)	0.0650879	0.034136026	1.906721189	0.056556702	871.7484501	ZIP	50
Johngarthia lagostoma	Ilha do Meio	lam(landscapeCover)gamma(.)p(moonPhase)iota(.)	det	(Intercept)	-0.7250688	0.145219222	-4.992925511	5.95E-07	871.7484501	ZIP	50
Johngarthia lagostoma	Ilha do Meio	lam(landscapeCover)gamma(.)p(moonPhase)iota(.)	det	scale(moonPhase)	0.099563	0.061981994	1.606321843	0.108203214	871.7484501	ZIP	50
Johngarthia lagostoma	Ilha do Meio	lam(landscapeCover)gamma(.)p(moonPhase)iota(.)	psi	psi	-1.7593309	0.479778738	-3.666963135	0.000245448	871.7484501	ZIP	50
Johngarthia lagostoma	Ilha do Meio	lam(.)gamma(TSE)p(moonPhase)iota(.)	lambda	(Intercept)	2.2575874	1.974955077	1.143108237	0.252993655	35540.15237	ZIP	50
Johngarthia lagostoma	Ilha do Meio	lam(.)gamma(TSE)p(moonPhase)iota(.)	gamma	(Intercept)	-0.5735142	NA	NA	NA	35540.15237	ZIP	50
Johngarthia lagostoma	Ilha do Meio	lam(.)gamma(TSE)p(moonPhase)iota(.)	gamma	scale(TSE)	-0.7464808	NA	NA	NA	35540.15237	ZIP	50
Johngarthia lagostoma	Ilha do Meio	lam(.)gamma(TSE)p(moonPhase)iota(.)	det	(Intercept)	0.5042346	54.50427575	0.009251286	0.992618647	35540.15237	ZIP	50
Johngarthia lagostoma	Ilha do Meio	lam(.)gamma(TSE)p(moonPhase)iota(.)	det	scale(moonPhase)	3.3107924	58.39257119	0.056698864	0.954785079	35540.15237	ZIP	50
Johngarthia lagostoma	Ilha do Meio	lam(.)gamma(TSE)p(moonPhase)iota(.)	psi	psi	-6.3434397	16.61212105	-0.381856096	0.702568107	35540.15237	ZIP	50
Johngarthia lagostoma	Ilha do Meio	lam(landscapeCover)gamma(TSE)p(moonPhase)iota(.)	lambda	(Intercept)	1.6352504	NA	NA	NA	35512.60276	ZIP	50
Johngarthia lagostoma	Ilha do Meio	lam(landscapeCover)gamma(TSE)p(moonPhase)iota(.)	lambda	landscapeCoveropen	0	NA	NA	NA	35512.60276	ZIP	50
Johngarthia lagostoma	Ilha do Meio	lam(landscapeCover)gamma(TSE)p(moonPhase)iota(.)	lambda	landscapeCovertreed	1.6692154	NA	NA	NA	35512.60276	ZIP	50
Johngarthia lagostoma	Ilha do Meio	lam(landscapeCover)gamma(TSE)p(moonPhase)iota(.)	gamma	(Intercept)	-0.3495322	NA	NA	NA	35512.60276	ZIP	50
Johngarthia lagostoma	Ilha do Meio	lam(landscapeCover)gamma(TSE)p(moonPhase)iota(.)	gamma	scale(TSE)	-0.4549738	NA	NA	NA	35512.60276	ZIP	50
Johngarthia lagostoma	Ilha do Meio	lam(landscapeCover)gamma(TSE)p(moonPhase)iota(.)	det	(Intercept)	1.1924796	NA	NA	NA	35512.60276	ZIP	50
Johngarthia lagostoma	Ilha do Meio	lam(landscapeCover)gamma(TSE)p(moonPhase)iota(.)	det	scale(moonPhase)	3.3073989	NA	NA	NA	35512.60276	ZIP	50
Johngarthia lagostoma	Ilha do Meio	lam(landscapeCover)gamma(TSE)p(moonPhase)iota(.)	psi	psi	-9.3845353	NA	NA	NA	35512.60276	ZIP	50
Johngarthia lagostoma	Ilha do Meio	lam(.)gamma(.)p(totalRainfallThreeMonths+moonPhase)iota(.)	lambda	(Intercept)	2.1969624	0.111416888	19.71839653	1.50E-86	771.0412653	ZIP	50
Johngarthia lagostoma	Ilha do Meio	lam(.)gamma(.)p(totalRainfallThreeMonths+moonPhase)iota(.)	gamma	(Intercept)	0.0914001	0.032029372	2.853633702	0.004322234	771.0412653	ZIP	50
Johngarthia lagostoma	Ilha do Meio	lam(.)gamma(.)p(totalRainfallThreeMonths+moonPhase)iota(.)	det	(Intercept)	-0.614958	0.163583308	-3.759295504	0.000170392	771.0412653	ZIP	50
Johngarthia lagostoma	Ilha do Meio	lam(.)gamma(.)p(totalRainfallThreeMonths+moonPhase)iota(.)	det	scale(totalRainfallThreeMonths)	0.9863806	0.104035481	9.481194129	2.51E-21	771.0412653	ZIP	50
Johngarthia lagostoma	Ilha do Meio	lam(.)gamma(.)p(totalRainfallThreeMonths+moonPhase)iota(.)	det	scale(moonPhase)	-0.455721	0.09327175	-4.88594839	1.03E-06	771.0412653	ZIP	50
Johngarthia lagostoma	Ilha do Meio	lam(.)gamma(.)p(totalRainfallThreeMonths+moonPhase)iota(.)	psi	psi	-1.9232533	0.544546778	-3.5318423	0.000412675	771.0412653	ZIP	50
Johngarthia lagostoma	Ilha do Meio	lam(landscapeCover)gamma(.)p(totalRainfallThreeMonths+moonPhase)iota(.)	lambda	(Intercept)	4.3066211	0.321849109	13.38086991	7.82E-41	672.0894835	ZIP	650
Johngarthia lagostoma	Ilha do Meio	lam(landscapeCover)gamma(.)p(totalRainfallThreeMonths+moonPhase)iota(.)	lambda	landscapeCoveropen	-0.4134047	0.212487064	-1.945552204	0.051708553	672.0894835	ZIP	650
Johngarthia lagostoma	Ilha do Meio	lam(landscapeCover)gamma(.)p(totalRainfallThreeMonths+moonPhase)iota(.)	lambda	landscapeCovertreed	1.316558	0.182576243	7.211003802	5.55E-13	672.0894835	ZIP	650
Johngarthia lagostoma	Ilha do Meio	lam(landscapeCover)gamma(.)p(totalRainfallThreeMonths+moonPhase)iota(.)	gamma	(Intercept)	0.0962644	0.024072823	3.998883019	6.36E-05	672.0894835	ZIP	650
Johngarthia lagostoma	Ilha do Meio	lam(landscapeCover)gamma(.)p(totalRainfallThreeMonths+moonPhase)iota(.)	det	(Intercept)	-3.8157082	0.288732242	-13.21538664	7.15E-40	672.0894835	ZIP	650
Johngarthia lagostoma	Ilha do Meio	lam(landscapeCover)gamma(.)p(totalRainfallThreeMonths+moonPhase)iota(.)	det	scale(totalRainfallThreeMonths)	0.7329663	0.060520576	12.11102689	9.23E-34	672.0894835	ZIP	650
Johngarthia lagostoma	Ilha do Meio	lam(landscapeCover)gamma(.)p(totalRainfallThreeMonths+moonPhase)iota(.)	det	scale(moonPhase)	-0.3939125	0.069716484	-5.650206588	1.60E-08	672.0894835	ZIP	650
Johngarthia lagostoma	Ilha do Meio	lam(landscapeCover)gamma(.)p(totalRainfallThreeMonths+moonPhase)iota(.)	psi	psi	-2.4683478	0.757209597	-3.259794623	0.001114929	672.0894835	ZIP	650
Johngarthia lagostoma	Ilha do Meio	lam(.)gamma(TSE)p(totalRainfallThreeMonths+moonPhase)iota(.)	lambda	(Intercept)	2.2424166	NA	NA	NA	35542.15006	ZIP	50
Johngarthia lagostoma	Ilha do Meio	lam(.)gamma(TSE)p(totalRainfallThreeMonths+moonPhase)iota(.)	gamma	(Intercept)	-0.5743338	217.1293998	-0.002645122	0.9978895	35542.15006	ZIP	50
Johngarthia lagostoma	Ilha do Meio	lam(.)gamma(TSE)p(totalRainfallThreeMonths+moonPhase)iota(.)	gamma	scale(TSE)	-0.7475549	166.8310951	-0.004480908	0.996424764	35542.15006	ZIP	50
Johngarthia lagostoma	Ilha do Meio	lam(.)gamma(TSE)p(totalRainfallThreeMonths+moonPhase)iota(.)	det	(Intercept)	1.4732953	NA	NA	NA	35542.15006	ZIP	50
Johngarthia lagostoma	Ilha do Meio	lam(.)gamma(TSE)p(totalRainfallThreeMonths+moonPhase)iota(.)	det	scale(totalRainfallThreeMonths)	2.3492419	NA	NA	NA	35542.15006	ZIP	50
Johngarthia lagostoma	Ilha do Meio	lam(.)gamma(TSE)p(totalRainfallThreeMonths+moonPhase)iota(.)	det	scale(moonPhase)	1.8923884	NA	NA	NA	35542.15006	ZIP	50
Johngarthia lagostoma	Ilha do Meio	lam(.)gamma(TSE)p(totalRainfallThreeMonths+moonPhase)iota(.)	psi	psi	-6.8418556	21.27537655	-0.32158564	0.747766624	35542.15006	ZIP	50
Johngarthia lagostoma	Ilha do Meio	lam(landscapeCover)gamma(TSE)p(totalRainfallThreeMonths+moonPhase)iota(.)	lambda	(Intercept)	1.6274711	NA	NA	NA	35514.60798	ZIP	50
Johngarthia lagostoma	Ilha do Meio	lam(landscapeCover)gamma(TSE)p(totalRainfallThreeMonths+moonPhase)iota(.)	lambda	landscapeCoveropen	0	NA	NA	NA	35514.60798	ZIP	50
Johngarthia lagostoma	Ilha do Meio	lam(landscapeCover)gamma(TSE)p(totalRainfallThreeMonths+moonPhase)iota(.)	lambda	landscapeCovertreed	1.6690047	NA	NA	NA	35514.60798	ZIP	50
Johngarthia lagostoma	Ilha do Meio	lam(landscapeCover)gamma(TSE)p(totalRainfallThreeMonths+moonPhase)iota(.)	gamma	(Intercept)	-0.3497328	NA	NA	NA	35514.60798	ZIP	50
Johngarthia lagostoma	Ilha do Meio	lam(landscapeCover)gamma(TSE)p(totalRainfallThreeMonths+moonPhase)iota(.)	gamma	scale(TSE)	-0.4552526	NA	NA	NA	35514.60798	ZIP	50
Johngarthia lagostoma	Ilha do Meio	lam(landscapeCover)gamma(TSE)p(totalRainfallThreeMonths+moonPhase)iota(.)	det	(Intercept)	2.1512072	NA	NA	NA	35514.60798	ZIP	50
Johngarthia lagostoma	Ilha do Meio	lam(landscapeCover)gamma(TSE)p(totalRainfallThreeMonths+moonPhase)iota(.)	det	scale(totalRainfallThreeMonths)	2.3777169	NA	NA	NA	35514.60798	ZIP	50
Johngarthia lagostoma	Ilha do Meio	lam(landscapeCover)gamma(TSE)p(totalRainfallThreeMonths+moonPhase)iota(.)	det	scale(moonPhase)	1.8593156	NA	NA	NA	35514.60798	ZIP	50
Johngarthia lagostoma	Ilha do Meio	lam(landscapeCover)gamma(TSE)p(totalRainfallThreeMonths+moonPhase)iota(.)	psi	psi	-9.978164	NA	NA	NA	35514.60798	ZIP	50
Sula dactylatra	Ilha do Meio	lam(.)gamma(.)p(.)iota(.)	lambda	(Intercept)	4.0222318	0.065462058	61.44371144	0	742.8925816	P	154
Sula dactylatra	Ilha do Meio	lam(.)gamma(.)p(.)iota(.)	gamma	(Intercept)	0.2095124	0.026805428	7.816043006	5.45E-15	742.8925816	P	154
Sula dactylatra	Ilha do Meio	lam(.)gamma(.)p(.)iota(.)	det	(Intercept)	1.2748729	0.179966917	7.083929475	1.40E-12	742.8925816	P	154
Sula dactylatra	Ilha do Meio	lam(.)gamma(TSE)p(.)iota(.)	lambda	(Intercept)	4.9148238	0.154350691	31.84192943	1.70E-222	598.8572812	P	754
Sula dactylatra	Ilha do Meio	lam(.)gamma(TSE)p(.)iota(.)	gamma	(Intercept)	0.2310945	0.024625393	9.384399055	6.33E-21	598.8572812	P	754
Sula dactylatra	Ilha do Meio	lam(.)gamma(TSE)p(.)iota(.)	gamma	scale(TSE)	0.1430325	0.038032598	3.760786859	0.00016938	598.8572812	P	754
Sula dactylatra	Ilha do Meio	lam(.)gamma(TSE)p(.)iota(.)	det	(Intercept)	-0.7957065	0.22121969	-3.596906521	0.000322024	598.8572812	P	754
Trachylepis atlantica	Ilha Rata	lam(.)gamma(.)p(.)iota(.)	lambda	(Intercept)	2.7918251	0.289755157	9.635117953	5.68E-22	304.7590824	ZIP	50
Trachylepis atlantica	Ilha Rata	lam(.)gamma(.)p(.)iota(.)	gamma	(Intercept)	0.1010307	0.056852758	1.777059105	0.075558516	304.7590824	ZIP	50
Trachylepis atlantica	Ilha Rata	lam(.)gamma(.)p(.)iota(.)	det	(Intercept)	-2.4144116	0.251286907	-9.60818708	7.38E-22	304.7590824	ZIP	50
Trachylepis atlantica	Ilha Rata	lam(.)gamma(.)p(.)iota(.)	psi	psi	-12.602251	121.907355	-0.103375643	0.917664843	304.7590824	ZIP	50
Trachylepis atlantica	Ilha Rata	lam(landscapeCover)gamma(.)p(.)iota(.)	lambda	(Intercept)	2.0718409	0.568706632	3.643075037	0.0002694	304.3818093	ZIP	50
Trachylepis atlantica	Ilha Rata	lam(landscapeCover)gamma(.)p(.)iota(.)	lambda	landscapeCoversemi-open	0.5379846	0.592813685	0.907510412	0.364136946	304.3818093	ZIP	50
Trachylepis atlantica	Ilha Rata	lam(landscapeCover)gamma(.)p(.)iota(.)	lambda	landscapeCovertreed	0.8576126	0.513145594	1.671285164	0.094665365	304.3818093	ZIP	50
Trachylepis atlantica	Ilha Rata	lam(landscapeCover)gamma(.)p(.)iota(.)	gamma	(Intercept)	0.0942811	0.056700405	1.662793606	0.096353757	304.3818093	ZIP	50
Trachylepis atlantica	Ilha Rata	lam(landscapeCover)gamma(.)p(.)iota(.)	det	(Intercept)	-2.4247506	0.236729507	-10.2427054	1.28E-24	304.3818093	ZIP	50
Trachylepis atlantica	Ilha Rata	lam(landscapeCover)gamma(.)p(.)iota(.)	psi	psi	-11.659091	76.07169844	-0.153264506	0.87818969	304.3818093	ZIP	50
Trachylepis atlantica	Ilha Rata	lam(.)gamma(.)p(observerID)iota(.)	lambda	(Intercept)	5.1991299	2.708121411	1.919828961	0.054879507	297.7525006	ZIP	450
Trachylepis atlantica	Ilha Rata	lam(.)gamma(.)p(observerID)iota(.)	gamma	(Intercept)	-0.0070377	0.080662599	-0.087248654	0.930473867	297.7525006	ZIP	450
Trachylepis atlantica	Ilha Rata	lam(.)gamma(.)p(observerID)iota(.)	det	(Intercept)	-5.2350274	2.730988247	-1.916898545	0.055250822	297.7525006	ZIP	450
Trachylepis atlantica	Ilha Rata	lam(.)gamma(.)p(observerID)iota(.)	det	observerIDMangini	0.7396644	0.309287844	2.391508187	0.01677931	297.7525006	ZIP	450
Trachylepis atlantica	Ilha Rata	lam(.)gamma(.)p(observerID)iota(.)	det	observerIDVini	0.7682435	0.407786039	1.883937708	0.059573402	297.7525006	ZIP	450
Trachylepis atlantica	Ilha Rata	lam(.)gamma(.)p(observerID)iota(.)	psi	psi	-5.6210138	3.729428827	-1.50720501	0.131758116	297.7525006	ZIP	450
Trachylepis atlantica	Ilha Rata	lam(landscapeCover)gamma(.)p(observerID)iota(.)	lambda	(Intercept)	2.62071	0.549634599	4.768095061	1.86E-06	301.2838486	ZIP	50
Trachylepis atlantica	Ilha Rata	lam(landscapeCover)gamma(.)p(observerID)iota(.)	lambda	landscapeCoversemi-open	0.4968096	0.542185587	0.916309125	0.359504783	301.2838486	ZIP	50
Trachylepis atlantica	Ilha Rata	lam(landscapeCover)gamma(.)p(observerID)iota(.)	lambda	landscapeCovertreed	0.7276314	0.472703643	1.539297249	0.123731745	301.2838486	ZIP	50
Trachylepis atlantica	Ilha Rata	lam(landscapeCover)gamma(.)p(observerID)iota(.)	gamma	(Intercept)	-0.0319105	0.073879006	-0.431929075	0.665792962	301.2838486	ZIP	50
Trachylepis atlantica	Ilha Rata	lam(landscapeCover)gamma(.)p(observerID)iota(.)	det	(Intercept)	-3.2285974	0.359033421	-8.992470418	2.42E-19	301.2838486	ZIP	50
Trachylepis atlantica	Ilha Rata	lam(landscapeCover)gamma(.)p(observerID)iota(.)	det	observerIDMangini	0.8074839	0.302866341	2.666139535	0.007672784	301.2838486	ZIP	50
Trachylepis atlantica	Ilha Rata	lam(landscapeCover)gamma(.)p(observerID)iota(.)	det	observerIDVini	0.8311281	0.428713304	1.93865718	0.052543096	301.2838486	ZIP	50
Trachylepis atlantica	Ilha Rata	lam(landscapeCover)gamma(.)p(observerID)iota(.)	psi	psi	-12.113229	95.46078506	-0.126892201	0.899025719	301.2838486	ZIP	50
Elaenia ridleyana	Ilha Rata	lam(.)gamma(.)p(.)iota(.)	lambda	(Intercept)	1.1238517	0.57735895	1.946538931	0.051590036	427.1494143	P	50
Elaenia ridleyana	Ilha Rata	lam(.)gamma(.)p(.)iota(.)	gamma	(Intercept)	0.0169584	0.127266935	0.13325092	0.893994942	427.1494143	P	50
Elaenia ridleyana	Ilha Rata	lam(.)gamma(.)p(.)iota(.)	det	(Intercept)	0.3109367	0.183957378	1.690265012	0.090977266	427.1494143	P	50
Elaenia ridleyana	Ilha Rata	lam(.)gamma(totalRainfallThreeMonths)p(.)iota(.)	lambda	(Intercept)	1.1291869	0.577491427	1.955330972	0.050544018	423.8833213	P	250
Elaenia ridleyana	Ilha Rata	lam(.)gamma(totalRainfallThreeMonths)p(.)iota(.)	gamma	(Intercept)	0.0179882	0.12812225	0.140398624	0.888345045	423.8833213	P	250
Elaenia ridleyana	Ilha Rata	lam(.)gamma(totalRainfallThreeMonths)p(.)iota(.)	gamma	scale(totalRainfallThreeMonths)	-0.3321267	0.148481214	-2.236826472	0.025297684	423.8833213	P	250
Elaenia ridleyana	Ilha Rata	lam(.)gamma(totalRainfallThreeMonths)p(.)iota(.)	det	(Intercept)	0.2799832	0.182060712	1.537856024	0.124083822	423.8833213	P	250
Johngarthia lagostoma	Ilha Rata	lam(.)gamma(.)p(.)iota(.)	lambda	(Intercept)	1.941561	0.18519991	10.48359568	1.03E-25	500.2593092	ZIP	50
Johngarthia lagostoma	Ilha Rata	lam(.)gamma(.)p(.)iota(.)	gamma	(Intercept)	0.2373139	0.039817794	5.95999723	2.52E-09	500.2593092	ZIP	50
Johngarthia lagostoma	Ilha Rata	lam(.)gamma(.)p(.)iota(.)	det	(Intercept)	-0.8450569	0.150396723	-5.618851964	1.92E-08	500.2593092	ZIP	50
Johngarthia lagostoma	Ilha Rata	lam(.)gamma(.)p(.)iota(.)	psi	psi	-1.3420531	0.610512701	-2.198239519	0.027932042	500.2593092	ZIP	50
Johngarthia lagostoma	Ilha Rata	lam(landscapeCover)gamma(.)p(.)iota(.)	lambda	(Intercept)	1.2206282	0.807035336	1.512484157	0.13041074	502.7424317	ZIP	50
Johngarthia lagostoma	Ilha Rata	lam(landscapeCover)gamma(.)p(.)iota(.)	lambda	landscapeCoversemi-open	0.618791	0.847624674	0.730029499	0.465372153	502.7424317	ZIP	50
Johngarthia lagostoma	Ilha Rata	lam(landscapeCover)gamma(.)p(.)iota(.)	lambda	landscapeCovertreed	0.802626	0.806506486	0.995188494	0.319644597	502.7424317	ZIP	50
Johngarthia lagostoma	Ilha Rata	lam(landscapeCover)gamma(.)p(.)iota(.)	gamma	(Intercept)	0.2370355	0.039885054	5.942964426	2.80E-09	502.7424317	ZIP	50
Johngarthia lagostoma	Ilha Rata	lam(landscapeCover)gamma(.)p(.)iota(.)	det	(Intercept)	-0.8403369	0.150308266	-5.590756182	2.26E-08	502.7424317	ZIP	50
Johngarthia lagostoma	Ilha Rata	lam(landscapeCover)gamma(.)p(.)iota(.)	psi	psi	-1.3161576	0.597494103	-2.202795961	0.027609133	502.7424317	ZIP	50
Johngarthia lagostoma	Ilha Rata	lam(.)gamma(.)p(totalRainfallThreeMonths)iota(.)	lambda	(Intercept)	1.9816309	0.189229567	10.47209975	1.16E-25	487.5915917	ZIP	50
Johngarthia lagostoma	Ilha Rata	lam(.)gamma(.)p(totalRainfallThreeMonths)iota(.)	gamma	(Intercept)	0.2049612	0.038772687	5.286226677	1.25E-07	487.5915917	ZIP	50
Johngarthia lagostoma	Ilha Rata	lam(.)gamma(.)p(totalRainfallThreeMonths)iota(.)	det	(Intercept)	-0.9532664	0.157960532	-6.034838917	1.59E-09	487.5915917	ZIP	50
Johngarthia lagostoma	Ilha Rata	lam(.)gamma(.)p(totalRainfallThreeMonths)iota(.)	det	scale(totalRainfallThreeMonths)	0.2843693	0.074965635	3.793328507	0.000148641	487.5915917	ZIP	50
Johngarthia lagostoma	Ilha Rata	lam(.)gamma(.)p(totalRainfallThreeMonths)iota(.)	psi	psi	-1.3736053	0.625683464	-2.195367771	0.028137227	487.5915917	ZIP	50
Johngarthia lagostoma	Ilha Rata	lam(landscapeCover)gamma(.)p(totalRainfallThreeMonths)iota(.)	lambda	(Intercept)	1.3056151	0.780627586	1.672519792	0.094421868	490.1961497	ZIP	50
Johngarthia lagostoma	Ilha Rata	lam(landscapeCover)gamma(.)p(totalRainfallThreeMonths)iota(.)	lambda	landscapeCoversemi-open	0.5811199	0.819104905	0.70945728	0.478040753	490.1961497	ZIP	50
Johngarthia lagostoma	Ilha Rata	lam(landscapeCover)gamma(.)p(totalRainfallThreeMonths)iota(.)	lambda	landscapeCovertreed	0.752441	0.777960588	0.967196853	0.333445635	490.1961497	ZIP	50
Johngarthia lagostoma	Ilha Rata	lam(landscapeCover)gamma(.)p(totalRainfallThreeMonths)iota(.)	gamma	(Intercept)	0.2048364	0.038840216	5.273821453	1.34E-07	490.1961497	ZIP	50
Johngarthia lagostoma	Ilha Rata	lam(landscapeCover)gamma(.)p(totalRainfallThreeMonths)iota(.)	det	(Intercept)	-0.9479756	0.158010462	-5.999448334	1.98E-09	490.1961497	ZIP	50
Johngarthia lagostoma	Ilha Rata	lam(landscapeCover)gamma(.)p(totalRainfallThreeMonths)iota(.)	det	scale(totalRainfallThreeMonths)	0.2834178	0.07503874	3.776952529	0.000158759	490.1961497	ZIP	50
Johngarthia lagostoma	Ilha Rata	lam(landscapeCover)gamma(.)p(totalRainfallThreeMonths)iota(.)	psi	psi	-1.3436903	0.610837787	-2.199749724	0.027824657	490.1961497	ZIP	50
Johngarthia lagostoma	Ilha Rata	lam(.)gamma(.)p(moonPhase)iota(.)	lambda	(Intercept)	1.9693808	0.184258202	10.68815812	1.16E-26	489.4278756	ZIP	50
Johngarthia lagostoma	Ilha Rata	lam(.)gamma(.)p(moonPhase)iota(.)	gamma	(Intercept)	0.2289282	0.039594028	5.781886418	7.39E-09	489.4278756	ZIP	50
Johngarthia lagostoma	Ilha Rata	lam(.)gamma(.)p(moonPhase)iota(.)	det	(Intercept)	-0.8370367	0.152248129	-5.497845512	3.84E-08	489.4278756	ZIP	50
Johngarthia lagostoma	Ilha Rata	lam(.)gamma(.)p(moonPhase)iota(.)	det	scale(moonPhase)	-0.258783	0.073570366	-3.51748953	0.00043565	489.4278756	ZIP	50
Johngarthia lagostoma	Ilha Rata	lam(.)gamma(.)p(moonPhase)iota(.)	psi	psi	-1.3527117	0.616517596	-2.194116902	0.028227006	489.4278756	ZIP	50
Johngarthia lagostoma	Ilha Rata	lam(landscapeCover)gamma(.)p(moonPhase)iota(.)	lambda	(Intercept)	1.2365879	0.808635909	1.529227085	0.126208158	491.8549176	ZIP	50
Johngarthia lagostoma	Ilha Rata	lam(landscapeCover)gamma(.)p(moonPhase)iota(.)	lambda	landscapeCoversemi-open	0.6279009	0.849354214	0.739268574	0.459743928	491.8549176	ZIP	50
Johngarthia lagostoma	Ilha Rata	lam(landscapeCover)gamma(.)p(moonPhase)iota(.)	lambda	landscapeCovertreed	0.8164691	0.808540024	1.009806644	0.312587936	491.8549176	ZIP	50
Johngarthia lagostoma	Ilha Rata	lam(landscapeCover)gamma(.)p(moonPhase)iota(.)	gamma	(Intercept)	0.228608	0.039670597	5.762655767	8.28E-09	491.8549176	ZIP	50
Johngarthia lagostoma	Ilha Rata	lam(landscapeCover)gamma(.)p(moonPhase)iota(.)	det	(Intercept)	-0.831632	0.152270339	-5.461549398	4.72E-08	491.8549176	ZIP	50

Johngarthia lagostoma	Ilha Rata	lam(landscapeCover)gamma(.)p(moonPhase)iota(.)	det	scale(moonPhase)	-0.2597547	0.073665438	-3.52614045	0.000421663	491.8549176	ZIP	50
Johngarthia lagostoma	Ilha Rata	lam(landscapeCover)gamma(.)p(moonPhase)iota(.)	psi	psi	-1.3248426	0.601938462	-2.20096022	0.02773884	491.8549176	ZIP	50
Johngarthia lagostoma	Ilha Rata	lam(.)gamma(.)p(totalRainfallThreeMonths+moonPhase)iota(.)	lambda	(Intercept)	2.6827392	0.299122669	8.968692351	3.00E-19	471.8630591	ZIP	450
Johngarthia lagostoma	Ilha Rata	lam(.)gamma(.)p(totalRainfallThreeMonths+moonPhase)iota(.)	gamma	(Intercept)	0.2415427	0.040055802	6.030154378	1.64E-09	471.8630591	ZIP	450
Johngarthia lagostoma	Ilha Rata	lam(.)gamma(.)p(totalRainfallThreeMonths+moonPhase)iota(.)	det	(Intercept)	-1.8531442	0.335903532	-5.516894163	3.45E-08	471.8630591	ZIP	450
Johngarthia lagostoma	Ilha Rata	lam(.)gamma(.)p(totalRainfallThreeMonths+moonPhase)iota(.)	det	scale(totalRainfallThreeMonths)	0.1303628	0.073026619	1.785140488	0.074238532	471.8630591	ZIP	450
Johngarthia lagostoma	Ilha Rata	lam(.)gamma(.)p(totalRainfallThreeMonths+moonPhase)iota(.)	det	scale(moonPhase)	-0.1731437	0.068048692	-2.54440914	0.010946279	471.8630591	ZIP	450
Johngarthia lagostoma	Ilha Rata	lam(.)gamma(.)p(totalRainfallThreeMonths+moonPhase)iota(.)	psi	psi	-1.3259164	0.600379076	-2.208465348	0.027211851	471.8630591	ZIP	450
Johngarthia lagostoma	Ilha Rata	lam(landscapeCover)gamma(.)p(totalRainfallThreeMonths+moonPhase)iota(.)	lambda	(Intercept)	1.3492312	0.776537725	1.737495986	0.082299662	485.4374728	ZIP	50
Johngarthia lagostoma	Ilha Rata	lam(landscapeCover)gamma(.)p(totalRainfallThreeMonths+moonPhase)iota(.)	lambda	landscapeCoversemi-open	0.5786705	0.814014037	0.710885178	0.477155391	485.4374728	ZIP	50
Johngarthia lagostoma	Ilha Rata	lam(landscapeCover)gamma(.)p(totalRainfallThreeMonths+moonPhase)iota(.)	lambda	landscapeCovertreed	0.7594396	0.772925367	0.982552338	0.325827812	485.4374728	ZIP	50
Johngarthia lagostoma	Ilha Rata	lam(landscapeCover)gamma(.)p(totalRainfallThreeMonths+moonPhase)iota(.)	gamma	(Intercept)	0.1981607	0.039670717	4.995137738	5.88E-07	485.4374728	ZIP	50
Johngarthia lagostoma	Ilha Rata	lam(landscapeCover)gamma(.)p(totalRainfallThreeMonths+moonPhase)iota(.)	det	(Intercept)	-0.9376826	0.160911449	-5.827320351	5.63E-09	485.4374728	ZIP	50
Johngarthia lagostoma	Ilha Rata	lam(landscapeCover)gamma(.)p(totalRainfallThreeMonths+moonPhase)iota(.)	det	scale(totalRainfallThreeMonths)	0.2317969	0.07921748	2.926082241	0.003432602	485.4374728	ZIP	50
Johngarthia lagostoma	Ilha Rata	lam(landscapeCover)gamma(.)p(totalRainfallThreeMonths+moonPhase)iota(.)	det	scale(moonPhase)	-0.1969862	0.076342877	-2.580283073	0.009871936	485.4374728	ZIP	50
Johngarthia lagostoma	Ilha Rata	lam(landscapeCover)gamma(.)p(totalRainfallThreeMonths+moonPhase)iota(.)	psi	psi	-1.3400332	0.609034298	-2.20025906	0.02778852	485.4374728	ZIP	50
Sula dactylatra	Ilha Rata	lam(.)gamma(.)p(.)iota(.)	lambda	(Intercept)	6.7642016	0.116118809	58.25241967	0	147.4353747	NB	2319
Sula dactylatra	Ilha Rata	lam(.)gamma(.)p(.)iota(.)	gamma	(Intercept)	0.3185825	0.035815987	8.89498079	5.84E-19	147.4353747	NB	2319
Sula dactylatra	Ilha Rata	lam(.)gamma(.)p(.)iota(.)	det	(Intercept)	-1.9891193	0.089925071	-22.11974077	2.04E-108	147.4353747	NB	2319
Sula dactylatra	Ilha Rata	lam(.)gamma(.)p(.)iota(.)	alpha	alpha	12.097749	32.56221796	0.371527167	0.710244925	147.4353747	NB	2319
