# Supplementary material for: Lessons from an eradication under multiple constraints of an island rat population of record density
Source: Conserv Biol. 2025 Dec 2;40(2):e70186. doi: 10.1111/cobi.70186 (PMC13036298; doi:10.1111/cobi.70186)
Supplement: Supplementary file 4 — Supporting Information [file COBI-40-e70186-s001.pdf]

Appendix S4 – Lessons from an eradication under multiple constraints of an island rat population of record density

Micheletti et al., 2025. Conservation Biology.

Table 1d. Results of a literature search conducted using the Scholarcy (https://www.scholarcy.com/) tool with the search string: *((("adaptive") AND ("management")) AND ("invasive")) AND ("species")*. The search aimed to identify studies that applied adaptive management in the context of invasive species. Each entry provides key details about the study, such as title, author, year of publication, whether adaptive management was used, whether the study was conducted on an island, if it was in a tropical setting, and whether it followed the six adaptive management points outlined by Westgate et al. (2013). Additionally, the Digital Object Identifier (DOI) for each study is listed for reference.

Title	Author	Year of publication	Uses AM?	Done on Island?	Tropical?	Follows Westgate 6 points?	DOI
**Successful recovery of North Island kokako Callaeas cinerea wilsoni populations, by adaptive management**	Innes, John	1999	Yes	No	No	1 to 5	10.1016/S0006-3207(98)00053-6
**Large scale predator control improves the productivity of a rare New Zealand riverine duck**	Whitehead, Amy L.	2008	Yes	No	No	1 to 5	10.1016/j.biocon.2008.08.013
**Energy and water use by invasive goats (Capra hircus) in an Australian rangeland, and a caution against using broad-scale allometry to predict species-specific requirements.**	Munn, A. J.	2012	No				10.1016/j.cbpa.2011.10.027
**A battle lost? Report on two centuries of invasion and management of Lantana camara L. in Australia, India and South Africa.**	Bhagwat, Shonil A.	2012	No				10.1371/journal.pone.0032407
**Mutual interactions between an invasive bark beetle and its associated fungi.**	Wang, B.	2012	No				10.1017/S000748531100037X
**Combining structured decision making and value-of-information analyses to identify robust management strategies.**	Moore, Joslin L.	2012	No				10.1111/j.1523-1739.2012.01907.x
**Anthropogenically induced adaptation to invade (AIAI): contemporary adaptation to**	Hufbauer, Ruth A.	2012	No				10.1111/j.1752-4571.2011.00211.x

**human-altered habitats within the native range can promote invasions.**							
**Climate-Driven Reshuffling of Species and Genes: Potential Conservation Roles for Species Translocations and Recombinant Hybrid Genotypes.**	Scriber, Jon Mark	2013	No				10.3390/insects5010001
**Climate change expands the spatial extent and duration of preferred thermal habitat for lake Superior fishes.**	Cline, Timothy J.	2013	No				10.1371/journal.pone.0062279
**Genomic patterns of introgression in rainbow and westslope cutthroat trout illuminated by overlapping paired-end RAD sequencing.**	Hohenlohe, Paul A.	2013	No				10.1111/mec.12239
**Nutrient limitation of native and invasive N2-fixing plants in northwest prairies.**	Thorpe, Andrea S.	2013	No				10.1371/journal.pone.0084593
**Migration and dispersal may drive to high genetic variation and significant genetic mixing: the case of two agriculturally important, continental hoverflies (Episyrphus balteatus and Sphaerophoria scripta).**	Raymond, Lucie	2013	No				10.1111/mec.12483
**Towards the planning and design of disturbance patterns across scales to counter biological invasions.**	Zurlini, Giovanni	2013	No				10.1016/j.jenvman.2013.05.006
**High Variation in Single Nucleotide Polymorphisms (SNPs) and Insertions/Deletions (Indels) in the Highly Invasive Bemisia tabaci (Gennadius) (Hemiptera: Aleyrodidae) Middle East-Asia Minor 1 (MEAM1).**	Lü, Z. C.	2013	No				10.1007/s13744-013-0152-2
**Novel microsatellite DNA markers indicate strict parthenogenesis and few genotypes in the invasive willow sawfly Nematus oligospilus.**	Caron, V.	2013	No				10.1017/S0007485312000429
**Stress for invasion success? Temperature stress of preceding generations modifies the response to insecticide stress in an invasive pest insect.**	Piiroinen, Saija	2013	No				10.1111/eva.12001

**Marsh thistle in New York: early detection and rapid response to a recent invader.**	Hinchey, Eamonn	2013	No					10.1111/nyas.12176
**The role of genomics in conservation and reproductive sciences.**	Johnson, Warren E.	2014	No					10.1007/978-1-4939-0820-2_5
**Increased survival and prolonged longevity mainly contribute to the temperature-adaptive evolutionary strategy in invasive Bemisia tabaci (Hemiptera: Aleyrodidae) Middle East Asia Minor 1.**	Lü, Zhi-Chuang	2014	No					10.1093/jisesa/ieu005
**Spinosad and the tomato borer Tuta absoluta: a bioinsecticide, an invasive pest threat, and high insecticide resistance.**	Campos, Mateus R.	2014	No					10.1371/journal.pone.0103235
**Learning from conservation planning for the U.S. National Wildlife Refuges.**	Meretsky, Vicky J.	2014	No					10.1111/cobi.12292
**Phenology research for natural resource management in the United States.**	Enquist, Carolyn A. F.	2014	No					10.1007/s00484-013-0772-6
**Adaptive responses reveal contemporary and future ecotypes in a desert shrub.**	Richardson, Bryce A.	2014	No					10.1890/13-0587.1
**A mid-term analysis of progress toward international biodiversity targets.**	Tittensor, Derek P.	2014	No					10.1126/science.1257484
**Priority threat management of invasive animals to protect biodiversity under climate change.**	Firn, Jennifer	2015	No					10.1111/gcb.13034
**Immune Reconstitution After Allogeneic Hematopoietic Stem Cell Transplantation and Association With Occurrence and Outcome of Invasive Aspergillosis.**	Stuehler, Claudia	2015	No					10.1093/infdis/jiv143
**The potential for biodiversity offsetting to fund effective invasive species control.**	Norton, David A.	2015	No					10.1111/cobi.12345
**Non-native megaherbivores: the case for novel function to manage plant invasions on islands.**	Hansen, Dennis M.	2015	No					10.1093/aobpla/plv085
**Intraspecific Niche Variation Drives Abundance-Occupancy Relationships in Freshwater Fish Communities.**	Faulks, Leanne	2015	No					10.1086/682004

**Consequences of seed origin and biological invasion for early establishment in restoration of a North American grass species.**	Herget, Mollie E.	2015	No				10.1371/journal.pone.0119889
**The whole genome sequence of the Mediterranean fruit fly, Ceratitis capitata (Wiedemann), reveals insights into the biology and adaptive evolution of a highly invasive pest species.**	Papanicolaou, Alexie	2016	No				10.1186/s13059-016-1049-2
**Adaptive management of invasive pests in natural protected areas: the case of Matsucoccus feytaudi in Central Italy.**	Sciarretta, A.	2016	Yes	No	No	Yes, but not all were specifically highlighted (i.e., point 2 is not specified -- management options). Note: eradication failed.	10.1017/S0007485315000851
**Biological invasions, ecological resilience and adaptive governance.**	Chaffin, Brian C.	2016	No				10.1016/j.jenvman.2016.04.040
**Targeted gene flow for conservation.**	Kelly, Ella	2016	No				10.1111/cobi.12623
**Diagnostic of Fungal Infections Related to Biofilms.**	Sanguinetti, Maurizio	2016	No				10.1007/5584_2016_9
**Improving credibility and transparency of conservation impact evaluations through the partial identification approach.**	McConnachie, Matthew M.	2016	No				10.1111/cobi.12610
**Evidence of Subdivisions on Evolutionary Timescales in a Large, Declining Marsupial Distributed across a Phylogeographic Barrier.**	Alpers, Deryn L.	2016	No				10.1371/journal.pone.0162789
**Controlling range expansion in habitat networks by adaptively targeting source populations.**	Hock, Karlo	2016	No				10.1111/cobi.12665
**Traditional Mapuche ecological knowledge in Patagonia, Argentina: fishes and other living beings inhabiting continental waters, as a reflection of processes of change.**	Aigo, Juana	2016	No				10.1186/s13002-016-0130-y

**Enhancing the effectiveness of biological control programs of invasive species through a more comprehensive pest management approach.**	DiTomaso, Joseph M.	2017	No				10.1002/ps.4347
**Erratum to: The whole genome sequence of the Mediterranean fruit fly, Ceratitis capitata (Wiedemann), reveals insights into the biology and adaptive evolution of a highly invasive pest species.**	Papanicolaou, Alexie	2017	No				10.1186/s13059-017-1155-9
**A quick and robust MHC typing method for free-ranging and captive primate species.**	de Groot, N.	2017	No				10.1007/s00251-016-0968-0
**Selection of invasive wild pig countermeasures using multicriteria decision analysis.**	Brondum, Matthew C.	2017	No				10.1016/j.scitotenv.2016.09.155
**Eradicating the grey squirrel Sciurus carolinensis from urban areas: an innovative decision-making approach based on lessons learnt in Italy.**	La Morgia, Valentina	2017	No				10.1002/ps.4352
**GMDPtoolbox: A Matlab library for designing spatial management policies. Application to the long-term collective management of an airborne disease.**	Cros, Marie-Josée	2017	No				10.1371/journal.pone.0186014
**Using Landscape Genetics Simulations for Planting Blister Rust Resistant Whitebark Pine in the US Northern Rocky Mountains.**	Landguth, Erin L.	2017	No				10.3389/fgene.2017.00009
**How to improve threatened species management: An Australian perspective.**	Scheele, B. C.	2018	No				10.1016/j.jenvman.2018.06.084
**Resilience and Adaptation: Yukon River Watershed Contaminant Risk Indicators.**	Duffy, Lawrence	2018	No				10.1155/2018/8421513
**High-Throughput Analysis Reveals Seasonal Variation of the Gut Microbiota Composition Within Forest Musk Deer (Moschus berezovskii).**	Hu, Xiaolong	2018	No				10.3389/fmicb.2018.01674
**Modelling the invasion history of Sinanodonta woodiana in Europe: Tracking the routes of a sedentary**	Konečný, Adam	2018	No				10.1111/eva.12700

**aquatic invader with mobile parasitic larvae.**							
**The role of invasive alien species in shaping local livelihoods and human well-being: A review.**	Shackleton, Ross T.	2019	No				10.1016/j.jenvman.2018.05.007
**Recent research status of Bactrocera dorsalis: Insights from resistance mechanisms and population structure.**	Wei, Dan-Dan	2019	No				10.1002/arch.21601
**Parallel introgression and selection on introduced alleles in a native species.**	Bay, Rachael A.	2019	No				10.1111/mec.15097
**Stress in captive Blue-fronted parrots (Amazona aestiva): the animalists' tale.**	Vidal, Alan Chesna	2019	No				10.1093/conphys/coz097
**A Novel Cold-Adaptive Endo-1,4-β-Glucanase From Burkholderia pyrrocinia JK-SH007: Gene Expression and Characterization of the Enzyme and Mode of Action.**	Chen, Feifei	2019	No				10.3389/fmicb.2019.03137
**Targeted gene flow and rapid adaptation in an endangered marsupial.**	Kelly, Ella	2019	No				10.1111/cobi.13149
**Enhancement of oxidative stress contributes to increased pathogenicity of the invasive pine wood nematode.**	Zhang, Wei	2019	No				10.1098/rstb.2018.0323
**Invasion origin, rapid population expansion, and the lack of genetic structure of cotton bollworm (Helicoverpa armigera) in the Americas.**	Gonçalves, Rogério Martins	2019	No				10.1002/ece3.5123
**Rapid growth and defence evolution following multiple introductions.**	van Boheemen, Lotte A.	2019	No				10.1002/ece3.5275
**Risk-Based and Adaptive Invasive Mosquito Surveillance at Lucky Bamboo and Used Tire Importers in the Netherlands.**	Ibáñez-Justicia, Adolfo	2020	No				10.2987/20-6914.1
**Integrated Methods for Monitoring the Invasive Potential and Management of Heracleum mantegazzianum (giant hogweed) in Switzerland.**	Shackleton, Ross T.	2020	No				10.1007/s00267-020-01282-9

**Testing the adaptive advantage of a threatened species over an invasive species using a stochastic population model.**	Brown, Timothy R.	2020	No				10.1016/j.jenvman.2020.110524
**Our Wild Companions: Domestic cats in the Anthropocene.**	Crowley, Sarah L.	2020	No				10.1016/j.tree.2020.01.008
**Abundance of invasive grasses is dependent on fire regime and climatic conditions in tropical savannas.**	Damasceno, Gabriella	2020	No				10.1016/j.jenvman.2020.111016
**Forest genomics: Advancing climate adaptation, forest health, productivity, and conservation.**	Isabel, Nathalie	2020	No				10.1111/eva.12902
**Reticulate evolution as a management challenge: Patterns of admixture with phylogenetic distance in endemic fishes of western North America.**	Bangs, Max R.	2020	No				10.1111/eva.13042
**Hitchhikers on floats to Arctic freshwater: Private aviation and recreation loss from aquatic invasion.**	Schwoerer, Tobias	2020	No				10.1007/s13280-019-01295-7
**Origin of resistance to pyrethroids in the redlegged earth mite (Halotydeus destructor) in Australia: repeated local evolution and migration.**	Yang, Qiong	2020	No				10.1002/ps.5538
**Dramatic long-term restoration of an oak woodland due to multiple, sustained management treatments.**	Glennemeier, Karen	2020	No				10.1371/journal.pone.0241061
**Spatial optimization of invasive species control informed by management practices.**	Nishimoto, Makoto	2021	No				10.1002/eap.2261
**Kentucky Bluegrass Invasion in the Northern Great Plains and Prospective Management Approaches to Mitigate Its Spread.**	Palit, Rakhi	2021	No				10.3390/plants10040817
**Fishery reforms for the management of non-indigenous species.**	Kleitou, Periklis	2021	No				10.1016/j.jenvman.2020.111690
**What Are the Keys to the Adaptive Success of European Wild Rabbit (Oryctolagus cuniculus) in the Iberian Peninsula?**	Marín-García, Pablo Jesús	2021	No				10.3390/ani11082453

**Adaptive population structure shifts in invasive parasitic mites, Varroa destructor.**	Moro, Arrigo	2021	No				10.1002/ece3.7272
**Rapid genetic adaptation to recently colonized environments is driven by genes underlying life history traits.**	Yin, Xiaoshen	2021	No				10.1186/s12864-021-07553-x
**The Impact of Climate Change on Forest Development: A Sustainable Approach to Management Models Applied to Mediterranean-Type Climate Regions.**	Nunes, Leonel J. R.	2021	No				10.3390/plants11010069
**Raiders of the last ark: the impacts of feral cats on small mammals in Tasmanian forest ecosystems.**	Lazenby, B. T.	2021	No				10.1002/eap.2362
**Genomic analysis unveils mechanisms of northward invasion and signatures of plateau adaptation in the Asian house rat.**	Chen, Yi	2021	No				10.1111/mec.16194
**Evolutionary genomics of endangered Hawaiian tree snails (Achatinellidae: Achatinellinae) for conservation of adaptive capacity.**	Price, Melissa R.	2021	No				10.7717/peerj.10993
**Prolonged Treatment with Grains of Paradise (Aframomum melegueta) Extract Recruits Adaptive Thermogenesis and Reduces Body Fat in Humans with Low Brown Fat Activity.**	Yoneshiro, Takeshi	2021	No				10.3177/jnsv.67.99
**Population genetic structure of raccoons as a consequence of multiple introductions and range expansion in the Boso Peninsula, Japan.**	Hirose, Miki	2021	No				10.1038/s41598-021-98029-1
**Risk management recommendations for environmental releases of gene drive modified insects.**	Devos, Yann	2022	No				10.1016/j.biotechadv.2021.107807
**The Prediction of Distribution of the Invasive Fallopia Taxa in Slovakia.**	Gašparovičová, Petra	2022	No				10.3390/plants11111484
**Vegetation, water infiltration, and soil carbon response to Adaptive Multi-Paddock and Conventional grazing in Southeastern USA ranches.**	Apfelbaum, Steven I.	2022	No				10.1016/j.jenvman.2022.114576

**Effectiveness of front line and emerging fungal disease prevention and control interventions and opportunities to address appropriate eco-sustainable solutions.**	Garvey, Mary	2022	No				10.1016/j.scitotenv.2022.158284
**Effect of microfibers combined with UV-B and drought on plant community.**	Deng, Jiaojiao	2022	No				10.1016/j.chemosphere.2021.132413
**(Epi)genomic adaptation driven by fine geographical scale environmental heterogeneity after recent biological invasions.**	Chen, Yiyong	2022	No				10.1002/eap.2772
**Applying early warning indicators to predict critical transitions in a lake undergoing multiple changes.**	Rohde, Elizabeth	2022	No				10.1002/eap.2685
**Habitat suitability and connectivity modeling predict genetic population structure and priority control areas for invasive nutria (Myocastor coypus) in a temperate river basin.**	Kang, Wanmo	2022	No				10.1371/journal.pone.0279082
**Historical museum samples enable the examination of divergent and parallel evolution during invasion.**	Stuart, Katarina C.	2022	No				10.1111/mec.16353
**Impact of roadside burning on genetic diversity in a high-biomass invasive grass.**	Di, Binyin	2022	No				10.1111/eva.13369
**Individual and population-scale carbon and nitrogen isotopic values of Procambarusclarkii in invaded freshwater ecosystems.**	Di Muri, Cristina	2022	No				10.3897/BDJ.10.e94411
**Native species exhibit physiological habituation to invaders: a reason for hope.**	Santicchia, Francesca	2022	No				10.1098/rspb.2022.1022
**Phenotypes and environment predict seedling survival for seven co-occurring Great Basin plant taxa growing with invasive grass.**	Agneray, Alison C.	2022	No				10.1002/ece3.8870
**Adaptive risk-based targeted surveillance for foreign animal diseases at the wildlife-livestock interface.**	Miller, Ryan S.	2022	No				10.1111/tbed.14576
**Remodeling the bladder tumor immune microenvironment by**	Senserrich, Jordi	2022	No				10.3389/fimmu.2022.993401

**mycobacterial species with changes in their cell envelope composition.**							
**Resistance Bioassays and Allele Characterization Inform Analysis of Spodoptera frugiperda (Lepidoptera: Noctuidae) Introduction Pathways in Asia and Australia.**	Tay, W. T.	2022	No				10.1093/jee/toac151
**The Role of Intestinal Microbial Metabolites in the Immunity of Equine Animals Infected With Horse Botflies.**	Hu, Dini	2022	No				10.3389/fvets.2022.832062
**Chromosome-level genome assembly and population genomic analyses provide insights into adaptive evolution of the red turpentine beetle, Dendroctonus valens.**	Liu, Zhudong	2022	No				10.1186/s12915-022-01388-y
**Are really Nature-Based Solutions sustainable solutions to design future cities in a context of global change? Discussion about the vulnerability of these new solutions and their probable unsustainable implementation.**	Duffaut, Chloé	2022	No				10.1016/j.scitotenv.2022.158535
**Evaluation of thermoregulation of different pine organs in early spring and estimation of heat reward for the western conifer seed bug (Leptoglossus occidentalis) on male cones.**	Kitajima, Ryotaro	2022	No				10.1371/journal.pone.0272565
**The high invasion success of fall armyworm is related to life-history strategies across a range of stressful temperatures.**	Wu, Pengxiang	2022	No				10.1002/ps.6867
**COI Haplotyping and Comparative Microbiomics of the Peach Fruit Fly, an Emerging Pest of Egyptian Olive Orchards.**	Awad, Mona	2022	No				10.3390/biology12010027
**Adaptive ecological knowledge among the Ndjuka Maroons of French Guiana; a case study of two 'invasive species': Melaleuca quinquenervia and Acacia mangium.**	Theys, Johanna	2023	No				10.1186/s13002-023-00602-7

**Adaptive evolution to the natural and anthropogenic environment in a global invasive crop pest, the cotton bollworm.**	Jin, Minghui	2023	No				10.1016/j.xinn.2023.100454	
**Disgust in animals and the application of disease avoidance to wildlife management and conservation.**	Sarabian, Cécile	2023	No				10.1111/1365-2656.13903	
**Open water dreissenid mussel control projects: lessons learned from a retrospective analysis.**	Dahlberg, Angelique D.	2023	No				10.1038/s41598-023-36522-5	
**Exploring vulnerabilities of inland fisheries in Indian context with special reference to climate change and their mitigation and adaptation: a review.**	Paul, Thankam Theresa	2023	No				10.1007/s00484-022-02417-9	
**Preadapted to adapt: underpinnings of adaptive plasticity revealed by the downy brome genome.**	Revolinski, Samuel R.	2023	No				10.1038/s42003-023-04620-9	
**Gene family expansion analysis and identification of the histone family in Spodoptera frugiperda.**	Gao, Han	2023	No				10.1016/j.cbd.2023.101142	
**Rapidly evolved traits enable new conservation tools: perspectives from the cane toad invasion of Australia.**	Shine, Richard	2023	No				10.1093/evolut/qpad102	
**Intraspecific trait plasticity to N and P of the wetland invader, Alternanthera philoxeroides under flooded conditions.**	Harms, Nathan E.	2023	No				10.1002/ece3.9966	
**The Invasive Caucasian Populations of the Brown Marmorated Stink Bug Halyomorpha halys (Hemiptera: Heteroptera: Pentatomidae) Rapidly Adapt Their Ecophysiological Traits to the Local Environmental Conditions.**	Reznik, Sergey Ya	2023	No				10.3390/insects14050424	
**Incorporating adaptive genomic variation into predictive models for invasion risk assessment.**	Chen, Yiyong	2024	No				10.1016/j.ese.2023.100299	
**Adaptive Management in EBIPM: A Key to Success in Invasive Plant Management**	Leffler	2012	No				10.2111/RANGELANDS-D-12-00053.1	

**Applied evolutionary biology could aid management of invaded ecosystems**	Oduor	2015	No					10.1890/EHS14-0026.1
**Adaptive management improves decisions about where to search for invasive species**	Rout	2017	No					10.1016/j.biocon.2017.04.009
**Adaptive invasive species distribution models: a framework for modeling incipient invasions**	Uden	2015	No					10.1007/s10530-015-0914-3

REFERENCE

Westgate MJ, Likens GE, Lindenmayer DB. 2013. Adaptive management of biological systems: A review. Biological Conservation 158:128–139.
